# Regions of low gene expression promote maintenance and adaptation of horizontally acquired genes in yeasts

**DOI:** 10.1038/s42003-026-10153-8

**Published:** 2026-05-09

**Authors:** Patrícia H. Brito, Victoria Gil, Ana Pontes, Margarida Silva, Carla Gonçalves, Paula Gonçalves

**Affiliations:** 1 Associate Laboratory i4HB-Institute for Health and Bioeconomy, NOVA School of Science and Technology, Universidade NOVA de Lisboa, Caparica, Portugal; 2 UCIBIO-Applied Molecular Biosciences Unit, Department of Life Sciences, NOVA School of Science and Technology, Universidade NOVA de Lisboa, Caparica, Portugal

**Keywords:** Molecular evolution, Fungal genomics, Fungal evolution

## Abstract

We showed previously that yeasts in *Starmerella* and *Wickerhamiella* genera (W/S clade) exhibit numbers of horizontal gene transfer (HGT) events much larger than found in any other yeast species, with bacteria and other fungi (Pezizomycotina) as donors. Here we shed light on HGT in the W/S clade by carrying out the characterization of xenologous genes present in three species harboring together close to 600 xenologous genes. Metabolic genes were strongly overrepresented and either introduced functions new to yeasts or restored functions that were likely absent in the W/S clade ancestor. RNA-sequencing revealed lower global levels of expression of xenologous *vs* native yeast genes in two species. This difference is associated with the preferential accumulation of xenologous genes in large, AT-enriched chromosome terminal regions, dubbed “End” domains, characterized by overall lower gene expression. In one species, “End” domains were shown to be more permissive for protein diversification, but only for xenologous genes. We posit that “End” domains may generate favorable conditions for the adaptation and retention of xenologous genes, helping explain the exceptional numbers of HGT events in the W/S clade.

## Abstract (web summary)

Horizontal gene transfer is generally considered uncommon in eukaryotes, but rates are very high in a floral yeast clade. Underlying this may be large, low-expression, chromosomal safe harbors that promote retention and adaptation of incoming genes.

## Introduction

Yeasts (*Saccharomycotina*) are ideal organisms for studying eukaryotic evolutionary biology due to their small genomes, straightforward phenotypic profiling, well-documented sexual reproduction systems, and the genetic tractability of numerous species. Moreover, the wealth of genomic data on yeasts collected over the past decade^1,2^ has significantly advanced our understanding of evolutionary processes in this sub-phylum. Important findings include the emergence of genetic code alterations^3,4^, the role of adaptive gene loss^5^, and the impact of horizontal gene transfer (HGT) and polyploidy on adaptation to both anthropogenic^6,7^ and natural environments^8^.

The clade comprising the *Wickerhamiella* and *Starmerella* yeast genera (henceforth the W/S-clade) harbors an order of magnitude more horizontally acquired genes than any other yeast lineage^1,9^. The clade includes nearly 100 species, many of which are yet to be formally described, and its speciose nature may result from adaptive radiation related to an hypothesized transition to the floral/floricolous insect niche^8^. As a result of research conducted by us^9–11^ and by others^12,13^ it became clear that horizontally acquired genes in the W/S-clade had multiple origins in both bacteria^1,9,10^ and filamentous fungi (*Pezizomycotina*)^11^. The first instance of HGT to be characterized in the W/S clade concerned the *FFZ1* gene, which encodes a high-capacity fructose transporter that underlies the fructophilic phenotype exhibited by an important fraction of W/S-clade species^11^. *FFZ1* was inferred to be absent in the yeast (*Saccharomycotina*) most recent common ancestor (MRCA), with its presence in the W/S clade attributed to horizontal acquisition from the Pezzizomycotina^11^. Subsequent analyses of the W/S-clade genomes available^1,9^, revealed that the evolutionary history of the W/S-clade seemed to include surges of HGT events preceded by massive gene loss in the MRCA of the clade, a pattern likely associated with the proposed ecological niche transition. This substantial loss of genes in the MRCA of the W/S clade was brought to light by the work of Shen et al.^1^, where all W/S clade genomes examined exhibited relatively low numbers of complete genes as assessed by BUSCO. We previously examined in detail a paradigmatic example of apparent functional replacement of yeast orthologues by bacterial genes, namely, the loss of alcoholic fermentation by the W/S-clade ancestor and its subsequent re-instatement in the *Starmerella* subclade, through the acquisition of a bacterial alcohol dehydrogenase^9^. Particularly interesting was also the discovery of two horizontal operon transfers (HOT) by us^10^ and by others^13^. In the first case, the optimization of a thiamine salvage pathway reconstructed from bacterial operons was refined by the independent horizontal acquisition of additional genes that expanded the range of assimilable thiamine derivatives^10^. In the second case, pertaining to iron scavenging, the acquisition of a fungal transporter for the siderophore enterobactin^12^, was apparently followed by the acquisition of a bacterial enterobactin biosynthetic operon^13^.

One topic that merits particular attention in the context of HGT is the adaptation of gene expression and regulation to the new host, especially in cases of inter-domain transfer. The study of the two HOT instances mentioned above has shed light on evolutionary paths for adaptation of polycistronic bacterial transcription to eukaryotic-style gene expression^10,13^. However, a general overview of the extent to which gene expression has adapted across large cohorts of horizontally acquired genes in the W/S clade—or, to our knowledge, in any other eukaryotic lineage—remains lacking. Such an overview represents a crucial first step toward understanding the tempo and mode of gene expression and regulatory adaptation following HGT in eukaryotes. The W/S clade provides an ideal system in which to address this gap through transcriptomic analyses because of the number of xenologous genes available for study and the time frame of the postulated HGT events.

We recently established a solid phylogenomic framework onto which several previously identified, well-characterized HGT events were mapped^14^. Our findings were in line with the initial hypotheses that these HGT events occurred either in the MRCA of the W/S-clade or in the MRCAs of the four main lineages within the clade^13,14^. This means that each gene acquired in one of these ancient HGT events has either been lost during evolution or is found in extant W/S-clade species, having evolved independently for some time during which functional adaptation to the new host occurred. Given the numbers of genes and species involved, this represents a wealth of information illuminating the evolutionary processes following HGT. This framework can now be used to address more in-depth the inner workings of the adaptation process and of the impact of xenologous genes on the biology of the yeast hosts. Fundamental questions still open concern the functional roles of the xenologous genes, their adaptive potential, transcriptional levels and genome localization and, most importantly, the biological determinants of the relative high success rates of HGT in the W/S-clade.

To address these questions, here we first established xenologous gene cohorts present in the genomes of three selected species. To a cohort of bacterial genes previously identified^9^ using the “alien index”^15,16^, genes originating in the *Pezizomycotina* were added, identified using a newly developed workflow. We then examined their functional roles, levels and patterns of expression and chromosomal localization. While the metabolic roles of most genes horizontally acquired by W/S-clade species consisted in the reinstatement of previously lost functions, an almost equal number of genes brought in new functions. Global levels of expression determined using RNA sequencing (RNA-seq) revealed species-specific patterns, with xenologous genes having lower average expression levels than native genes in two species. Lower expression of xenologous genes turned out to be linked to their presence in terminal chromosomal regions with relatively lower gene content and lower overall levels of gene expression. The exceptional length of these regions (up to 600 kb), which we named “end” domains, and their AT-enrichment are characteristics that distinguish them from subtelomeric regions. We also found that, unlike native genes, xenologous genes in “end” domains have significantly higher evolutionary rates as measured by the ratio of non-synonymous to synonymous substitution rates (d*N*/d*S*). Altogether, we conclude that the characteristics of “end” domains are likely to have contributed to facilitate the integration, retention, and adaptation of foreign genes in W/S-clade species.

## Results

### Establishing xenologous gene datasets

**Fig. 1:**
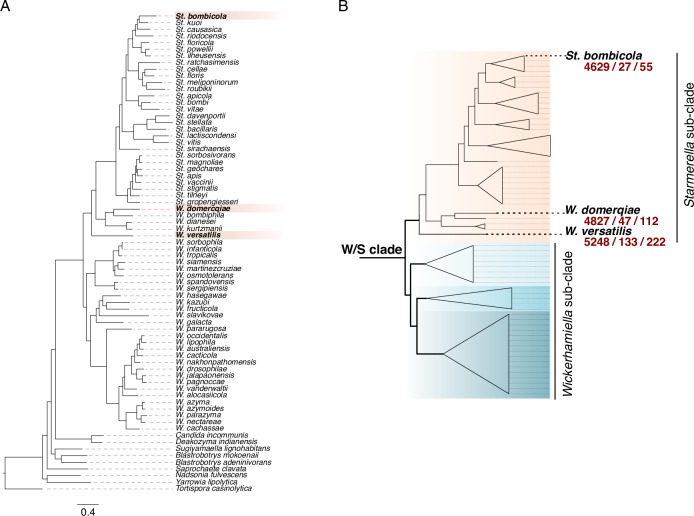
Phylogenetic relationships of the W/S-clade species. **A** Maximum-likelihood phylogenomic analysis, including 63 W/S-clade species and nine outgroup species. **B** Simplified W/S clade phylogeny, highlighting the three focal species and sub-clades within the W/S clade. The *Starmerella* sub-clade is shown in salmon, while the *Wickerhamiella* sub-clade is highlighted in blue. The total number of native and xenologous genes per species are indicated in red underneath each focal species name as follows: native/horizontally acquired from filamentous fungi (*Pezizomycotina*)/horizontally acquired from bacteria. Original data for total gene origins are in Supplementary Data 6–8.

Our characterization of HGT focused on three W/S-clade species, for which we obtained chromosome-level genome assemblies: *Wickerhamiella versatilis*, *Wickerhamiella domercqiae,* and *Starmerella bombicola* (Fig. 1A). The first two species have the largest numbers of horizontally acquired genes among species examined so far ref. ^9^, and the third is the model species within the clade and the only one for which molecular genetic tools are available^9^. For clarity, it should be noted that in spite of having been classified in the *Wickerhamiella* genus, phylogenomic analyses clarified that *W. versatilis* and *W. domercqiae* unequivocally belong in the *Starmerella* sub-clade of the W/S-clade tree^1,8,14^ (Fig. 1A, B).

Genes originating in bacteria were previously identified in these three species^9^ using the “alien index” pipeline^15,16^. Identification of genes originating in the filamentous fungi (*Pezizomycotina*) required the development of a classifier to sort them out from native genes, a task complicated by the phylogenetic proximity between yeasts and filamentous fungi. Our analysis relies on the comparison of the distribution of gene-tree distance and relative frequencies of homologous gene copies determined in different phylogenetic groups to identify the best HGT candidates (Figs. S1–S3). We further used manually curated phylogenetic analyses to confirm the origin of candidate orthologues in the *Pezizomycotina*. The results obtained with the new classifier are shown in detail in Supplementary Data 1–5. The genomes of *W. versatilis, W. domercqiae,* and *St. bombicola* were found to contain 133, 47, and 27 genes from *Pezizomycotina*, respectively, which is about half the number of genes from bacteria that were identified on those genomes (Fig. 1B). Throughout this work, we use the term “native gene” to designate genes for which the evolutionary origin was neither in the *Pezizomycotina* nor in bacteria. In very few cases (<20), we detected putative HGT events appearing to originate in the Basidiomycota that were not further studied because the proteome dataset used in the classifier was not tailored to confidently identify HGT events originating in the Basidiomycota. These genes were not removed from the native gene cohort.

Multiple lines of evidence suggest that W/S-clade HGTs did not resulted from recent transference events. Previously, an important fraction of the genes of bacterial origin was found in more than one W/S-clade species^9,14^. Specifically, 52 orthologous groups were found in at least two of the seven species examined, and their evolutionary histories corroborate the species tree. This suggested that many genes were acquired by the ancestors of extant species. In addition to this phylogenomic evidence, the relative antiquity of the W/S-clade HGTs (originating both in bacteria and in the *Pezizomycotina*) was corroborated in all cases presently investigated by the fact that no close match (>70% identity) was observed between protein sequences encoded by acquired genes and putative donor sequences available in public databases.

### Functions of horizontally acquired genes in *W. versatilis, W. domercqiae,* and *St. bombicola*

**Fig. 2:**
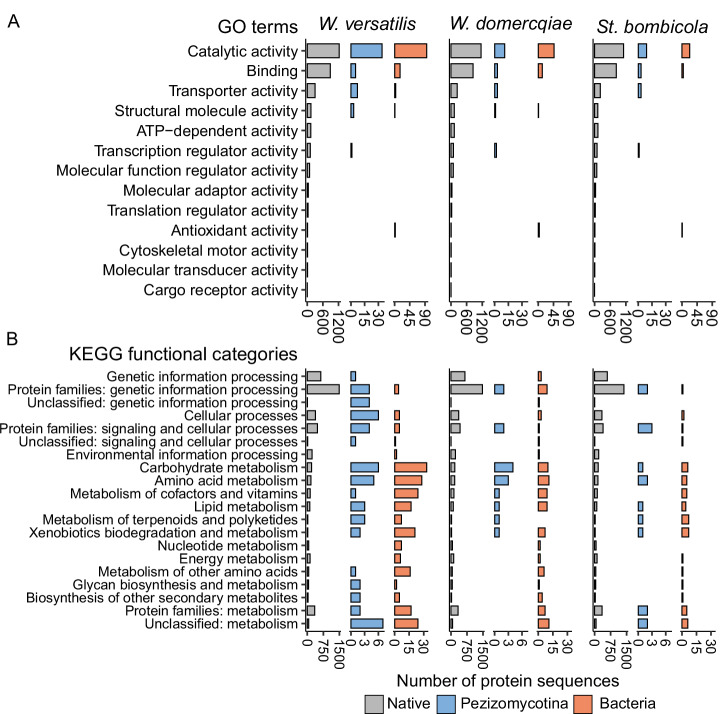
Functional characterization of W/S-clade genomes by gene origin. **A** Assignment of xenologous and native genes to gene ontology categories using PANTHER GO-slim molecular functions **B** KEGG functional classification of xenologous and native protein-coding sequences generated with KO assignments from BLAST-Koala, mapped to functional hierarchical categories of the KEGG BRITE database. Genes with KO assignments to “organismal systems”, “human diseases”, “poorly characterized”, and “without KO” are not included in this figure. Original data are in Supplementary Data 6–8 and 19.

To investigate the functional roles of xenologous genes, KEGG orthology (KO)^17^ identifiers were assigned to all genes and used to reconstruct KEGG pathways^18,19^. This pipeline successfully assigned a KO identifier to approximately half of the genes in each genome (Supplementary Data 6–8). Functional coverage was expanded using the PANTHER Classification System^20^, which assigned gene ontology (GO) terms and broad functional categories based on PANTHER GO-Slim and PANTHER Protein Class annotations (Supplementary Data 6–8). Combining KO and PANTHER annotations resulted in functional classifications for more than 85% of all genes across the three genomes. When considering xenologous genes only, functional information was assigned to 96.6% of the *W. versatilis* xenologous genes, 94.3% of the *W. domercqiae* xenologous genes and 98.8% of the *St. bombicola* xenologous genes (Supplementary Data 6–8). Most xenologous genes with inferred PANTHER protein classes were classified as metabolite interconversion enzymes (PC00262). Consistently, the most highly represented pathways within the three HGT cohorts are carbohydrate, vitamin, co-factor, and amino-acid metabolism (Figs. 2, S4, and Supplementary Data 9).

We also noted that molecular functions are not necessarily evenly distributed between the two xenologous gene cohorts: for example, while horizontally acquired transporters (PANTHER protein class PC00227) tend to originate from *Pezizomycotina*, xenologous metabolite interconversion enzymes (PANTHER protein class PC00262) are largely derived from bacteria (Supplementary Data 9). Specifically, Supplementary Data 9 shows that of the 24 xenologous transporter genes identified, 19 originated from *Pezizomycotina*. These observations are suggestive of increased difficulties in the operationalization of bacterial transporters in the eukaryotic host. In contrast, of the 352 acquired metabolite interconversion enzymes present in the three species, 284 were acquired from bacteria. Concerning non-metabolic functions, *W. versatilis* acquired six xenologous gene-specific transcription activators (PC00264; Supplementary Data 9), and the other two species each have one transcription activator gene, acquired from bacteria. Suggestive of some impact on cell-regulatory functions is the presence of a total of 24 proteins (15 in *W. versatilis)* in the category “protein modifying enzymes” (PC00260; Supplementary Data 9), a protein class that includes kinases, phosphatases and methylases, among others. Further improvement of functional annotation of the xenologous genes lacking both KO and PANTHER assignments was performed using the EggNOG-mapper^21^ and InterProScan^22,23^ pipelines. These additional analyses yielded functional information for seven previously unannotated xenologous genes in *W. versatilis* and *W. domercqiae* (Supplementary Data 10). Among these, two genes are involved in carbohydrate metabolism, three encode putative transcription factors, and two genes encode secreted fungal proteins of unknown function (Supplementary Data 10).

**Fig. 3:**
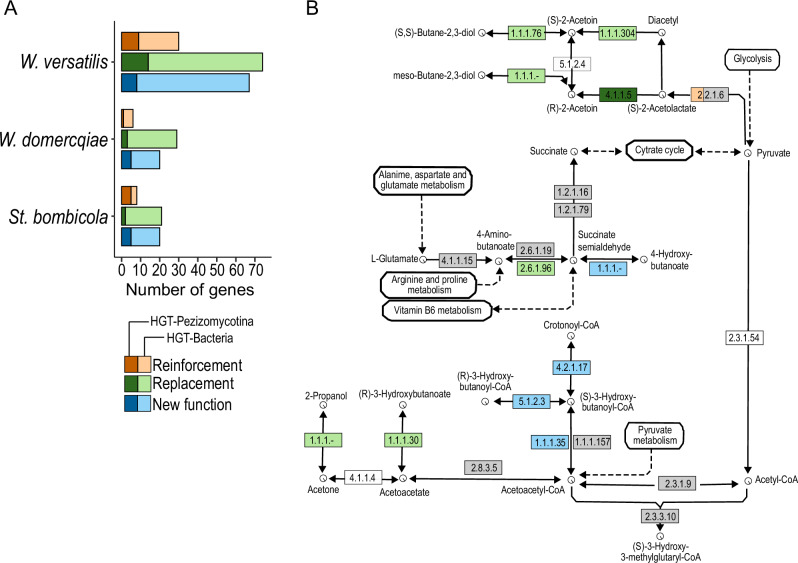
Contribution of horizontally acquired genes to the host genome. **A** The contribution of horizontally acquired genes to the host genome was determined by evaluating the presence of homologous genes and their functions within the *Saccharomycotina* subphylum. Classification of the HGT events as replacement of previously lost functions (green), new function (blue) or reinforcement (salmon) was conducted as explained in Fig. S5. Darker colors indicate genes acquired from filamentous fungi (*Pezizomycotina*) and lighter colors indicate genes acquired from bacteria. Original data are on Supplementary Data 6–8. **B** The butanoate metabolic pathway reconstructed for the *W. versatilis* genome using the KEGG mapper tool. Native genes are marked in gray, xenologous genes follow the color scheme of plot (**A**); genes absent in the genome of *W. versatilis* are marked in white.

We next used well-defined criteria (Fig. S5) to determine the fractions of functionally classified genes from each species that brought in entirely new functions to the host, those replacing a function previously lost in the ancestor of the clade^1^, and those that seem to be reinforcing functions fulfilled by native genes (Fig. 3A). The latter were a minority (29 genes in *W. versatilis*, 6 in *W. domercqiae* and 8 in *St. bombicola*). Replacement of previous lost functions (74 genes in *W. versatilis*, 28 in *W. domercqiae* and 21 in *St. bombicola*) seems to be the most frequent role of HGT events in all three species, followed closely by the acquisition of new functions (67 genes in *W. versatilis*, 20 in *W. domercqiae* and 20 in *St. bombicola*). Absence of a native ortholog in the focal species implied that the xenologous gene fulfilled one of the two latter roles (Fig. S5). The distinction between these two possibilities relied on detailed analysis of OrthoFinder-generated trees complemented by blast searches to support the decision on whether it was possible to find yeast orthologues with the same putative function of the xenologous gene in publicly available yeast genomes (Fig. S5). If no such orthologues could be found, the gene was considered to encode a function new to yeasts; otherwise, it was classified as a replacement. In Fig. 3B, an example is shown in which genes representing new functions, replacements and to a lesser degree reinforcements, of both bacterial and *Pezizomycotina* origin, are intertwined with native genes in metabolic pathways related with butane-2,3-diol metabolism.

### Determining global transcription patterns

Expression of horizontally acquired genes immediately after acquisition is expected to be sub-optimal due to the lack of appropriate promoters governing expression of incoming genes, while the process of adaptation is expected to impact levels and modes of expression. Hence, we subsequently set out to investigate how average expression levels of the large cohort of xenologous genes in the three W/S clade species under study compared to the average levels of expression of native genes in the same species.

**Fig. 4:**
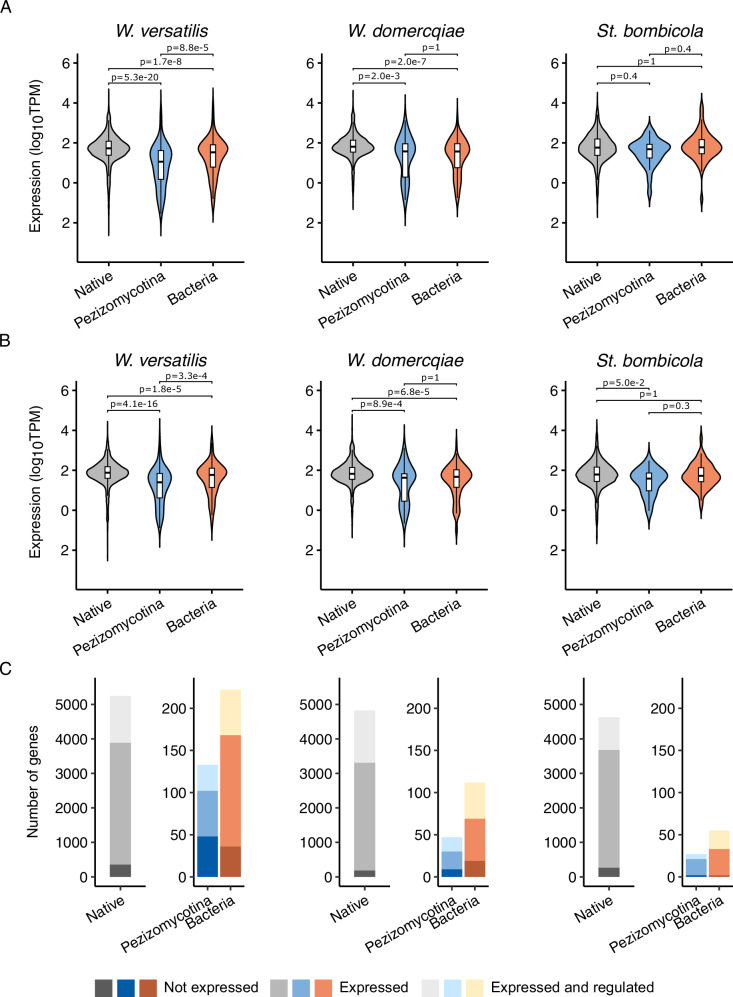
Comparative transcriptomics of W/S-clade species by gene origin. **A**, **B** Distribution of expression levels of vertically acquired genes (native, gray), and horizontally acquired genes from filamentous fungi (*Pezizomycotina*, blue), and bacteria (orange) for *W. versatilis*, *W. domercqiae,* and *St. bombicola*. Transcriptomes were obtained from cells cultivated in YPD (**A**), and YNB with 2% w/v glucose as carbon source (**B**), harvested in mid-exponential phase. Expression levels were measured in transcripts per million reads (TPM) and represent the mean expression across three biological replicates. Data were log₁₀-transformed prior to analysis and visualization. Statistical significance between expression profiles was assessed using the pairwise Mann–Whitney *U* test, with *p*-values adjusted using the Bonferroni correction for multiple comparisons. **C** Gene expression and regulation across W/S clade species by gene origin. For each group of genes, we present the total number of genes “not expressed”, “expressed”, and “expressed and regulated”. A gene was considered expressed when the average expression was equal or higher than 5 TPM in either YPD or YNB medium; genes differentially expressed between these two growth conditions (Fig. S6) were considered regulated. Original data are in Supplementary Data 7–9 and 19.

To that end, RNA-seq data was obtained for the three species cultivated in triplicate in two different growth conditions routinely used for yeast growth—YNB-based defined composition medium and YPD rich medium, both containing 2% w/v glucose as carbon source (Supplementary Data 11 and 12). This allowed us to determine global transcriptional levels of native and xenologous genes under both conditions (Supplementary Data 6–8). RNA sequencing datasets revealed species-specific, rather than condition-specific, patterns when comparing the global expression levels of native and both types of xenologous genes, as shown in Fig. 4A. Whereas in *St. bombicola,* global levels of expression of native, bacterial and *Pezizomycotina* genes are not significantly different under both growth conditions, in *W. versatilis* and in *W. domercqiae*, both types of xenologous genes are expressed on average at significantly lower levels (Fig. 4A, B). This is unlike reports in other unicellular eukaryotes, where global levels of expression of xenologous genes were indistinguishable from native genes^24^, possibly reflecting that W/S clade gene acquisitions are relatively more recent. In Fig. 4C, the relative numbers of unexpressed, expressed, and expressed-and-regulated genes are shown, considering as regulated genes those exhibiting significantly different expression levels in the two growth conditions tested (Fig. S6). Genes with TPM counts below 5 were considered not expressed, abiding by a criterion previously used for RNA-seq analyses in a W/S-clade species^13^. Whereas *St. bombicola* virtually lacks unexpressed genes of both xenologous origins (4% of the total), *W. domercqiae* and *W. versatilis*, harbor substantial fractions of xenologous genes for which no expression was observed in both growth conditions (38% and 23% of the total, respectively), in line with the significant differences of average transcriptional levels to native genes shown in Fig. 4A, B. Fisher’s exact test confirmed that the three species differ significantly in their proportion of expressed to non-expressed genes (*p* = 1.2 × 10^−4^). Post-hoc pairwise Fisher’s exact tests (Holm-adjusted) indicated that both *W. versatilis* and *W. domercqiae* differed significantly from *St. bombicola* (*p* = 1.5 × 10^−4^ and *p* = 9.8 × 10^−3^, respectively), whereas *W. versatilis* and *W. domercqiae* do not differ significantly from each other (*p* = 0.13). However, the fraction of unexpressed genes in *W. versatilis* likely also reflects the circumstance that only two growth conditions were tested. In fact, our analysis of RNA-seq data in the public domain, concerning the *W. versatilis* transcriptome in cells grown on YPD solid medium^13^, uncovered a few additional HGT genes expressed under those conditions, that were classified as unexpressed in our analysis (Supplementary Data 6).

**Fig. 5:**
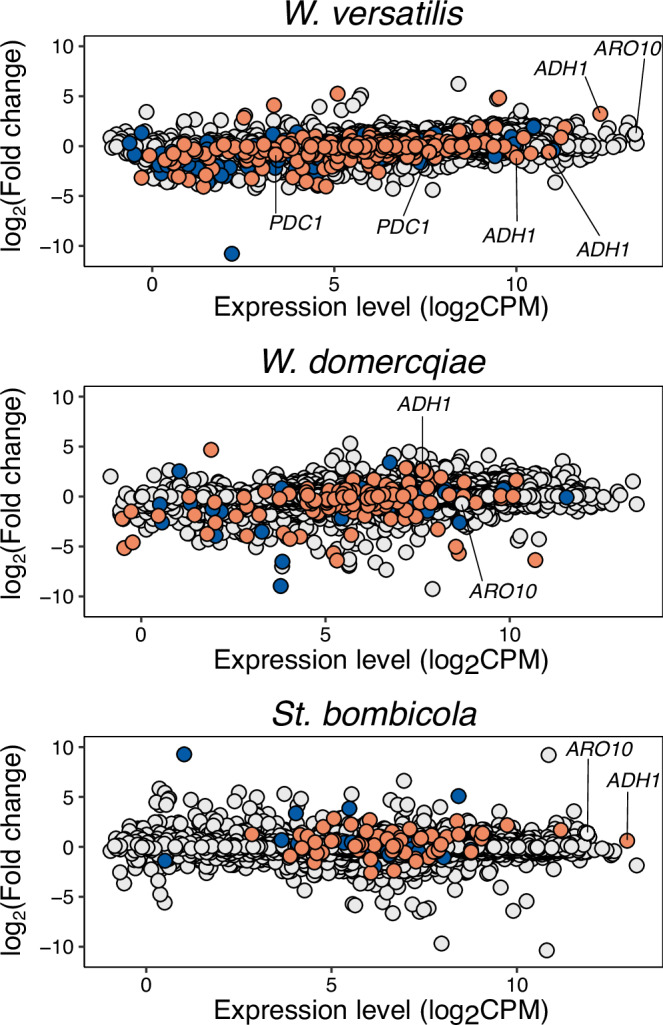
Gene expression levels of fermentative genes. Mean-difference (MD) plot showing average expression level of each gene (x-axis) and differential expression in the two growth conditions studied (y-axis). Genes are identified by their origin: native (gray), bacterial (orange) and *Pezizomycotina* (blue). Genes involved in alcoholic fermentation in *St. bombicola* (Gonçalves et al.^9^) and their homologues in the other two species are labeled, highlighting very high levels of expression for fermentation key genes in the two fermentative species (*St. bombicola* and *W. versatilis*), including an alcohol dehydrogenase gene of bacterial origin (*ADH1)*, when compared with their levels of expression in non-fermentative species *W. domercqiae*. On the contrary, *PDC1* genes of bacterial origin in *W. versatilis* have modest expression levels, suggesting that a co-opted native *ARO10* gene may fulfill the role of *PDC* in alcoholic fermentation, as previously demonstrated for *St. bombicola* (Gonçalves et al.^9^). Data on this figure [log₂(FC) and log₂(CPM)] were obtained after the differential expression analysis performed using edgeR; full data is provided on Supplementary Data 6–8 and 19.

Some horizontally acquired genes were among the most highly expressed. For example, in fermenting species *St. bombicola* and *W. versatilis* (bacterial) alcohol dehydrogenase genes of the type participating in alcoholic fermentation (Adh1-like) are very highly expressed (Supplementary Data 6–8 and Fig. 5). Accordingly, the native *ARO10* gene previously shown to have functionally replaced the first enzyme of the alcoholic fermentation pathway, *PDC*, in *St. bombicola*^9^, is also very highly expressed in these species (Fig. 5). On the other hand, in *W. domercqiae* (Fig. 5) a species that does not produce ethanol^25^, both the bacterial *ADH1* and native *ARO10* genes are expressed at lower levels (Fig. 5). Interestingly, *W. versatilis* is so far the only W/S-clade species to harbor two *PDC* genes of bacterial origin^9^. This could suggest that alcoholic fermentation in this species would rely exclusively on the bacterial *PDC* genes, bypassing the native *ARO10*. However, while *ARO10* is expressed in *W. versatilis* at a much higher rate than might be expected in connection with its normal role in amino acid catabolism, the bacterial *PDC* genes are expressed at an order of magnitude lower levels (Fig. 5). This observation raises questions about the functional relevance of bacterial *PDC* genes in alcoholic fermentation. Instead, it suggests that *ARO10* may have a role in *W. versatilis* similar to that fulfilled in *St. bombicola*. Taken together, these observations illustrate clear links between the expression of horizontally acquired genes and phenotype. They also demonstrate that bacterial genes may attain very high levels of expression upon adaptation to the yeast host.

### Chromosomal localization of xenologous genes versus expression

Chromosomal localization has been shown to have ample implications in gene function^26,27^. Nonessential genes and those exhibiting noisier transcription tend to localize to different parts of the eukaryotic chromosome from essential genes with low transcription noise^28^. The former include subtelomeric regions that typically also exhibit low gene density, low synteny, more sequence repeats and higher intraspecies variation in gene content^27,29^. Such regions were, in addition, proposed to be entry points for horizontally acquired genes in eukaryotes^30^. On the other hand, chromosome localization is known to affect transcriptional levels, like in the telomere position effect described first in *Saccharomyces cerevisiae*^31^.

**Fig. 6:**
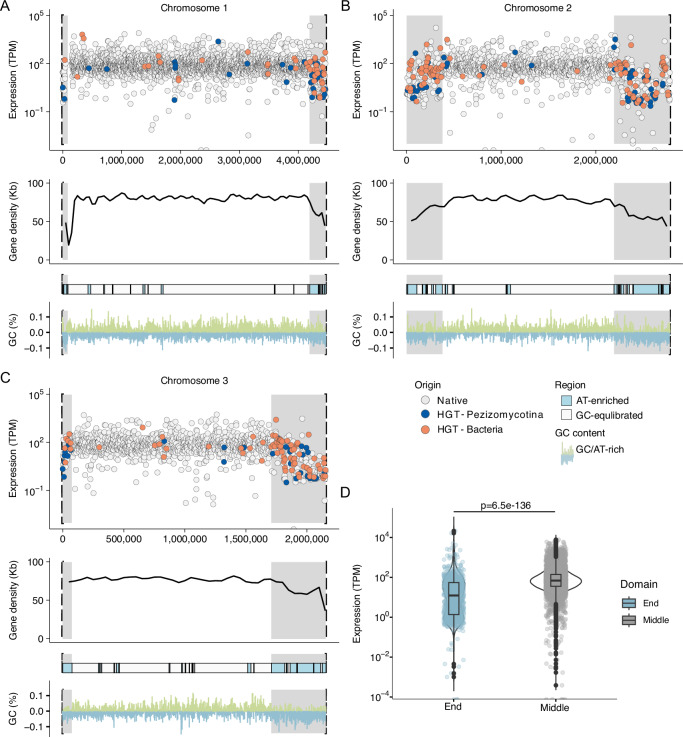
Transcription levels vs. chromosomal localization in *W. versatilis.* **A**, **B**, **C** Depict identical analyses for nuclear chromosomes 1, 2, and 3, respectively. The top plot in each panel shows, for the pertinent chromosome, the average transcription levels (y-axis) for native (gray) and horizontally acquired genes from *Pezizomycotina* (blue) and bacteria (orange) plotted against chromosomal localization (x-axis). Vertical dashed lines mark telomere positions. Middle plots show gene density along chromosomes calculated as total genome length occupied by protein-coding genes in 100 kb sliding windows. The horizontal bar shows the position of genomic regions classified as AT-rich (blue) or GC-equilibrated (white), by the software OcculterCut. Bottom panels display GC content as deviation from the genome average: GC-rich shown above (light green), and AT-rich below (light blue). Vertical gray bars at chromosome extremities mark the positions of “end” domains across all plots, determined based on the distribution of AT-rich regions identified with OcculterCut (full explanation of the determination of the localization and extent of “end” domains is given in Supplementary Data 13). **D** Violin plots depict average gene expression levels of all genes irrespective of origin (native and xenologous of both origins), within “end” (blue) and “middle” (gray) chromosomal domains. Statistical significance was assessed using the Mann–Whitney *U* test (*U* = 881880, *p* = 6.51 × 10^136^). For each gene, the average expression level considered was the mean transcription levels calculated from six RNA-seq data sets (YPD and YNB grown cells, three replicates for each condition) measured in transcript per million (TPM) and plotted on a log_10_ scale. Data include genes from all three chromosomes. Full data is provided in Supplementary Data 6 and 19.

To address these matters, we mapped the localization of xenologous and native genes onto chromosome-level genome assemblies of each species, generated with high-coverage Nanopore long reads, and polished with Illumina short reads. Each species harbors three large nuclear chromosomes (Supplementary Data 12 and Figs. 6, S7, S8), and telomere repeats were identified in nearly all chromosomal ends. The assemblies are of high quality, as indicated by contiguity metrics (N50: 2.58–3.22 Mb; coverage: 102x- 200x; Supplementary Data 12) and the presence of telomere repeats at most termini. To further evaluate completeness, we applied the BUSCO pipeline to quantify the percentage of complete, fragmented and missing BUSCO orthologs. Approximately 80% of BUSCO genes were classified as complete in all three assemblies (Supplementary Data 12), consistent with previous observations^1,2^. The relatively low percentage of BUSCO genes classified as complete reflects, as expected, the extensive gene loss that occurred in the ancestor of the W/S-clade lineage^1^. The maps obtained showed that, in *W. versatilis* and to a much lesser degree in *W. domercqiae*, acquired genes accumulate close to chromosome termini, while in *St. bombicola,* that is not evident (Fig. S9). The maps also plot for each gene its average level of expression across the six RNA-seq datasets available for each species (YPD and YNB grown cells, three independent cultures, Supplementary Data 6–8), bringing to light that close to chromosome termini both native and xenologous genes seem to have lower expression levels, an effect observed in all six extremities of the three *W. versatilis* nuclear chromosomes, albeit to different degrees, and in most of the *W. domercqiae* and *St. bombicola* chromosome ends. Low expression is the least pronounced at the left terminus of *W. versatilis* chromosome 1 and the left terminus of chromosome 2 in *St. bombicola*, where rDNA clusters are located. We then searched for additional chromosomal features in all three species, including the position of centromeres—which could not be determined with confidence - and genome-wide variation in GC content. The latter analysis revealed AT-enriched regions (deviation from genome average, Fig. 6A–C), mostly located close to chromosome termini, their distribution closely resembling that of the transcriptionally depressed regions. We subsequently employed a tool, OcculterCut^32^, designed to delimit AT-enriched/depleted chromosomal domains in fungal genomes. The output of this analysis predicted that ca. 20% of the *W. versatilis* genome and 5% of the *St. bombicola* genome consisted of AT-enriched domains (Figs. 6A–C and S8A–C, Supplementary Data 13), while for *W. domercqiae,* the software was unable to define a threshold on which to base the delimitation of domains with significantly different AT/GC content under the default settings. The AT-enriched domains determined by the OcculterCut software using the threshold values defined for *W. versatilis* and *St. bombicola* were mapped against the chromosomes. As seen in Fig. 6A–C for *W. versatilis* and in Fig. S8A–C for *St. bombicola*, and as expected based on the results for AT/GC content deviation from genome average (Figs. 6A–C and S8A–C), most AT-enriched domains defined by OcculterCut are localized close to chromosome ends in both species. Next, based on the OcculterCut results, we delimited chromosome “end” domains encompassing most AT-enriched domains in *W. versatilis* and *St. bombicola*, as shown in Figs. 6A–C and S8A–C), and we classified genes as being either within these “end” domains or outside these domains, in regions we dubbed “middle”, in the two species. A comparison between the average levels of expression in genes located within “end” and “middle” domains (Figs. 6D, S8D, and Supplementary Data 13) was performed next. The results, shown in Figs. 6D and S8D, confirm significantly lower levels of expression for genes located at “end” domains when compared to “middle” domains in both *W. versatilis* (Mann–Whitney *U* test, *U* = 881880, *p* = 6.51 × 10^−136^) and *St. bombicola* (Mann–Whitney *U* test, *U* = 7675.5, *p* = 7.6 × 10^−16^).

We next examined the genomic distribution of transposable elements (TEs) identified using the Earl Grey pipeline^33^ (Supplementary Data 14). The TEs identified were “unclassified”, indicating that they could not be assigned to known transposon families. The genomes of *W. versatilis* and *W. domercqiae* contain strikingly few TEs, representing approximately 0.2% and 1% of their genomes, respectively (Supplementary Data 14). Despite their low abundance, TEs in both species are predominantly localized in gene-poor regions, as commonly observed for eukaryotic transposons (Supplementary Data 14)^34^, and therefore most are found within or in the vicinity of “end” domains. In contrast, *St. bombicola* exhibits a markedly higher abundance and diversity of transposons, accounting for ~7% of the genome, with the highest densities found again near telomeres located within “end” domains. Consistent with the preferential localization of both TEs and xenologous genes toward chromosomal ends, the average distance between TEs and xenologous genes is significantly shorter than between TEs and native genes in *W. versatilis* and *W. domercqiae* (Mann–Whitney *U* test; *W. versatilisı*: *U* = 1387614, *p* = 3.4 × 10^−69^; *W. domercqiae*: *U* = 478575, *p* = 4.5 × 10^−9^; Supplementary Data 14). By contrast, this difference is much weaker in *St. bombicola* (Mann–Whitney *U* test, *U* = 214378, *p* = 3.5 × 10^−2^), consistent with the absence of significant accumulation of xenologous genes within “end” domains in this species (Fig. S9).

Together, these observations indicate that genome TE content is not positively correlated with the propensity to acquire or retain xenologous genes, as the most TE-rich species—*St. bombicola—* is also the one harboring the fewest xenologous genes. We also assessed the potential impact of TEs on the expression of neighboring genes, particularly xenologous genes. While in *W. domercqiae* gene expression was not significantly affected by the proximity of a TE, for the other two species, significant differences were observed (Supplementary Data 14). In *W. versatilis*, genes flanked (at 1 kb or less) upstream or downstream by a TE have significantly lower expression, but we found only 27 genes matching this criterium, which discards the vicinity of TEs as the source of generalized transcriptional repression in *W. versatilis* “end” domains. No genes were found that were flanked (up to 1 kb distance) upstream and downstream by TEs in this species. In *St. bombicola*, significantly lower expression was found for genes located closer than 1 kb to a TE in either direction, and for genes flanked up- and downstream by TEs. The latter cohort exhibited the lowest average expression levels.

Finally, using a combination of HMM-based protein searches^35,36^ and a dedicated starship detection pipeline^37^, we performed genome searches aiming to identify “starship transposons”, known to play a prominent role in HGT across the *Pezizomycotina*. These searches failed to identify any such TEs in the W/S-clade genomes available, in line with previous findings indicating that “starship transposons” are absent in yeast genomes^37^.

While, as mentioned above, low transcription regions close to telomeres also occur in other eukaryotes, the size of some of those regions in the W/S-clade species here examined is unusually large, in some cases more than 500 kb (*W. versatilis*, Supplementary Data 13). Regions exhibiting telomere position effect reported so far in other fungi range from a few kb to 100 kb in *Schizosaccharomyces pombe*^38^. To get some insight on whether this type of particularly extensive transcriptionally depressed region could be found outside the W/S clade, we plotted gene expression *vs* chromosome position for three species in the same order (Dipodascales) as the W/S clade and with increasing phylogenetic proximity—*Yarrowia lypolytica*, *Sugyamaella lignohabitans,* and *Candida incommunis*, the latter being very closely related to the W/S clade (Fig. S10A–N). For the first two, it was possible to find both long read assemblies and RNA-seq data available in the public domain^39,40^ (Supplementary Data 15), while for *C. incommunis,* we produced both long read genome assembly and RNA-seq data. As depicted in Supplementary Fig. S10, no large regions of depressed transcription were observed in any of these three species. Moreover, for the three Dipodascales species, we determined the variation of GC content along the chromosome by using OcculterCut to search for a threshold value between eventual AT-enriched/GC-equilibrated regions. For *Y. lypolytica,* it was not possible to determine such a threshold, but for the other two species, thresholds were found, and AT-enriched domains were identified and mapped onto chromosomes (Fig. S11). Unlike in the W/S clade, where AT-enriched domains were the minority and were located almost always close to chromosome ends, in *Su. lignohabitans* and *C. incommunis* AT-enriched regions dominated and were distributed across the chromosomes (Supplementary Data 16).

Finally, we also failed to detect the presence of large transcriptionally depressed domains in *Hanseniaspora uvarum* (Fig. S10O–U) that represents a distantly related lineage, but which, like the W/S clade^41^, is defective in cell cycle and DNA repair genes^42^. In this species, OcculterCut also failed to identify a threshold to guide the classification of genomic regions as AT-enriched (Supplementary Data 16).

In conclusion, we identified depressed transcription in telomere-adjacent chromosomal domains of the W/S clade (“end” domains). These domains are exceptionally long and AT-enriched, and may or maynot be enriched in TEs, distinguishing them from previously described subtelomeric regions. Analysis of yeast species outside the W/S clade failed to reveal comparable “end” domains. In *W. versatilis*, we further observed a significant accumulation of xenologous genes within these “end” domains.

### Evidence for relaxed purifying selection in *W. versatilis* chromosomal “end” domains

**Fig. 7:**
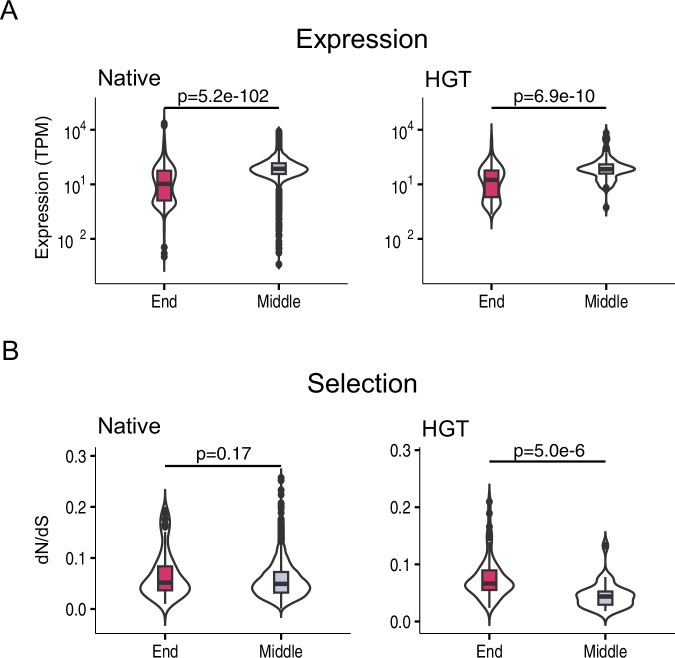
Evolutionary dynamics vs. expression of native and xenologous (HGT) genes in *W. versatilis* in “end” and “middle” chromosomal domains. **A** Average levels of expression for native and xenologous (HGT) genes (calculated from the six RNA-seq datasets produced for *W. versatilis*) located in either “end” or “middle” domains, showing that decreased expression levels affect both native and xenologous genes present in “end” domains. Statistical significance was assessed using the Mann–Whitney *U* test (native genes: *U* = 557470.5, *p* = 5.2 × 10^−102^; xenologous (HGT) genes: *U* = 5012, *p* = 6.9 × 10^−10^). **B** Average d*N*/d*S* ratio for genes in “end” and “middle” chromosomal domains, showing significantly higher evolution rates for xenologous genes at “end” domains but not for native genes similarly located. Statistical significance was assessed using the Mann–Whitney *U* test (native genes: *U* = 76362, *p* = 0.17; xenologous (HGT) genes: *U* = 716, *p* = 5.0 × 10^−6^). The data include genes from all three chromosomes. Full data is provided in Supplementary Data 19.

Subtelomeric regions were often found to be cradles of evolutionary innovation, which may also include the accumulation of mutations^29,43,44^. To assess this in *W. versatilis* “end” domains, we measured the nonsynonymous versus synonymous substitution ratio (d*N*/d*S*) of single-copy orthologs estimated in *W. versatilis* and a closely related *Wickerhamiella sp*. isolate that is 28% divergent at the nucleotide level. Single-copy orthologs were selected and subsequently classified according to their location in the *W. versatilis* genome in either “middle” or “end” domains. The rate of synonymous (dS) and nonsynonymous (dN) substitutions and the d*N*/d*S* ratio were determined using codeml implemented in PAML^45^. After exclusion of orthologs with dS values above 5, deemed to be too divergent to include in the analyses^46^, the d*N*/d*S* ratio was significantly higher at “end” domains than in “middle” chromosomal positions due to the mutational process occurring at xenologous genes (Mann–Whitney *U* test, *U* = 716, *p* = 5.0 × 10^−6^, Fig. 7). d*N*/d*S* values were nevertheless well below 1 with one exception (Fig. S12). Maximum-likelihood tests using either one d*N*/d*S* ratio (M0) or codon site models (M2a vs. M1a and M8 vs. M7) performed with codeml did not recover significant evidence for positive selection on the genes under study after Bonferroni correction of significant values (Supplementary Data 17). Gene-wise relative synonymous codon usage (gRSCU) was also very similar for genes at both chromosomal locations (Fig. S13), indicating the absence of a strong codon usage bias at “end” domains that could constrain dS and elevate d*N*/d*S* ratios. Hence, the patterns of nucleotide substitutions with increased d*N*/d*S* ratios that arose from this analysis are consistent with relaxed purifying selection of xenologous genes (i.e., increased tolerance for protein sequence variation) at “end” domains, when compared to “middle” domains.

## Discussion

The *Wickerhamiella/Starmerella* yeast clade is unique in the opportunities it offers for the study of HGT in unicellular eukaryotes, outside the realm of HGT linked to endosymbiosis, which is frequent in protists^30,47^. A recent global analysis concerning genes of prokaryotic origin in fungi estimated an average number of 16 genes per genome in the Ascomycota^48^, which is much lower than the number of genes of prokaryotic origin in the species studied here. HGT is, thus, particularly pervasive in the W/S clade and specifically in the *Starmerella* sub-clade that includes all three species analyzed in this study (Fig. 1B). Incoming genes may have helped rapid adaptation to new niches, often at the interface between bees and flowers^49^. This includes the reconstruction of fermentative lifestyles previously lost in the W/S-clade ancestor^9,14^. Corroborating previous findings^10,11,13,14,50^, the HGT events found presently are not recent, since identity between the acquired genes and putative donor sequences remained always below 70%. This is consistent with phylogenetic evidence revealing HGT occurrences in the MRCA of the entire W/S-clade^13,14^ and others taking place later in the ancestors of each of the four main sub-clades (Fig. 1)^10,14^. The relative antiquity of the events constitutes an opportunity to reconstruct evolutionary trajectories of the genes after acquisition by the first yeast host. Also, the species radiation presumably fostered in the W/S-clade by xenologous gene acquisition and elevated mutation rates^41^ concurrent with niche transition, provides a rich phylogenetic context in which to observe the emergence of metabolic novelty, for example in alcoholic fermentation^14^, in the stepwise assembly of a bacterial operon-based thiamine salvage pathway^10^ and in the functional cooperation between a fungal (*Pezizomycotina*) transporter^12^ and an adapted bacterial operon^13^ to foster iron metabolism.

The similarity of the functional pathways and roles in which the W/S-clade HGTs are implicated, with those involved in HGT events in other eukaryotes, is striking^47,48,51–53^. In line with this, over 80% of the genes to which a function could be ascribed are metabolic enzymes, and their functions can be generally described as broadening the nutritional range and promoting competitive fitness in microbial communities. Illustrative of the latter is the presence of a gene encoding a lysozyme-family protein in the cohort of *W. versatilis* genes originating in the *Pezizomycotina* (Supplementary Data 6). Lysozyme (muramidase) enzymes constitute a defense against bacteria and have been frequently transferred horizontally across the tree of life^54^.

One important question we addressed while functionally classifying the HGT genes was their evolutionary role in present-day W/S-clade genomes, i.e., whether they brought entirely new functions to W/S-clade species or were replacing functions lost in the MRCA of the clade or even if they are reinforcing certain enzymatic steps. The latter case can be regarded as a gene copy number increase if the xenologous and native enzymes have similar activities but can also harbor some hidden innovation in case the incoming enzyme contributes with different kinetic parameters or substrate specificity. Although limited by the fact that functions in pathways could be attributed only to about half of the xenologous genes, our conclusion was that most genes were brought in to replace genes normally present in *Saccharomycotina* species but were lost in the MRCA of the W/S clade. A striking example of this is alcoholic fermentation, which was reconstructed in the *Starmerella* sub-clade thanks to the acquisition of a bacterial alcohol dehydrogenase^9^. However, an almost equal number of genes was associated with new functions.

Among the three W/S-clade species here studied in detail, *W. versatilis* harbors the most xenologous genes, 335 (6% of the total number of genes in this species). Since no recent acquisitions were identified, we posit that this species likely retained more xenologous genes after the ancient HGT surges than the sister lineage encompassing all the other known species in the *Starmerella* sub-clade (Fig. 1B). Both the absolute number of xenologous genes and the fraction of the genome they represent are large in comparison with other fungi [Dikarya, average of 27 genes per genome but with large variation between species; Ascomycota, 16 genes per genome^48^]. A comparison within the realm of unicellular eukaryotes shows that protists also have much lower HGT rates than the W/S-clade species studied here and even lower than fungi in general^55^. While the opportunities afforded by endosymbiosis, frequent in protists, should facilitate genetic interchange, it was recently proposed that the relatively low HGT rates in protists could be related to the decreased transferability of genes providing nutritional advantages in phagocytizing unicellular eukaryotes when compared to osmotrophic fungi^47^.

Horizontally acquired genes accumulate in *W. versatilis* in large (over 500 kb, Supplementary Data 13) AT-enriched domains at chromosome ends. Much shorter versions of such regions are also found in *St. bombicola* and *W. domercqiae*, although in the latter species this can only be ascertained from plots showing deviation of GC content from genome average (Fig. S7). We dubbed these “end” domains, not subtelomeric regions, because they were delimited resorting to genome wide differences in AT/GC content between chromosomal regions, while subtelomeric regions are usually delimited by gene density/essentiality^56^ and transposon/repeat presence^57^ close to chromosome ends. Subtelomeric regions and the “end” domains here defined have, however, features in common, notably low gene density and decreased gene expression. Because of their extension and the large number of xenologous genes they contained, we studied in more detail the “end” domains of *W. versatilis*, including the determination of protein evolution rates which was impossible in the other two species because either objective delimitation of AT-enriched domains (using the software OcculterCut) was unattainable—in *W. domercqiae*—precluding the definition of “end” domains in this species, or because xenologous gene numbers at “end” domains were too low (in *St. bombicola*). “End” domains exhibit significantly lower average levels of expression of both native and xenologous genes than genes located in “middle” domains. This is reminiscent of the telomere position effect first studied in *S. cerevisiae,* but here acting over much larger distances and is likely similarly caused by repressive chromatin structures propagating inwards from the telomeres^58^. The intensity of transcription repression in “end” domains tends to be inversely proportional to the distance to the telomeres, but with many exceptions, suggesting that the overall repressing effect is frequently overruled by gene-specific regulatory systems. It is possible that AT-enrichment in W/S-clade species “end” domains, which is not a characteristic previously reported in yeast subtelomeric regions, is due to the natural AT-bias of spontaneous mutations in eukaryotes, as observed in *S. cerevisiae*^59^, combined with the deficit in DNA repair-related genes reported for W/S clade species^41^. This is because, like transcription, DNA repair is negatively affected by less accessible chromatin^60^. Notably, proficient DNA repair promotes chromatin remodeling and accessibility, so that it is possible that the DNA repair deficit contributes to the exceptionally lengthy low transcription regions in *W. versatilis*, thus indirectly affecting both local AT/GC content and transcriptional levels over an unusual long range. Although the underlying causes of AT-enrichment in “end” domains cannot be established with confidence, it is possible to exclude some possibilities. For example, it emerged from our analysis that AT- enrichment in *W. versatilis* “end” domains was not caused by the accumulation of xenologous genes in these regions, since average AT-levels were significantly higher in native genes than in xenologous genes, both located in “end” domains (Supplementary Data 13). In fact, when the different cohorts of genes are compared, the largest difference in AT/GC content is between native genes in “end” domains and native genes in “middle” domains, the former exhibiting clearly significantly higher AT levels. (Mann–Whitney *U* test; *W. versatilis*: *U* = 675354, *p* = 2.2 × 10^−16^; *W. domercqiae*: *U* = 32726, *p* = 3.5 × 10^−5^; Supplementary Data 13). This concurs with our working hypothesis that “end” domains predated the horizontal acquisition of genes by W/S clade ancestors (discussed below) and is compatible with a role for defective DNA repair in AT-enrichment.

Why should low gene expression have a positive impact on HGT, as implied by our observations in *W. versatilis*? It is possible that the low expression levels observed in “end” domains may help mitigate potential expression-related costs of HGT, as observed in bacteria^61^, which seem to be mainly associated with the expression of maladapted proteins. Indeed, misfolding was proposed to be the main restrainer of evolution rates (d*N*/d*S*) in highly expressed genes^62^, and xenologous proteins are probably more likely than native proteins to have high rates of misfolding prior to adaptation to the new host. Hence, lower expression is expected to weaken selective pressure, leading to a relaxation of purifying selection (thus, higher evolution rates), which in turn increases the likelihood of xenologous genes adapting before being purged by selection. The observed increased tolerance to protein sequence evolution in the *W. versatilis* “end” domains, when compared to other chromosomal locations, was manifest in increased d*N*/d*S* ratios in comparison with “middle” chromosome domains and could help explain the persistence of large numbers of xenologous genes in these regions. In fact, xenologous genes at “end” domains show significantly higher evolutionary rates than when they are located at “middle” domains (Mann–Whitney *U* test, *U* = 716, *p* = 5.0 × 10^−6^), suggesting that the increase in d*N*/d*S* could have an underlying adaptive drive.

The evidence uncovered here affords a glimpse into possible reasons contributing to the unusual propensity of the ancestors of the W/S-clade to acquire and retain horizontally acquired genes. Our observations are aligned with the view that depressed transcription in “end” domains is not a consequence of the presence of large numbers of xenologous genes in W/S-clade species, because depressed transcription is observed also in chromosome termini where no high accumulation of xenologous genes is found, like in *St. bombicola*. Notably, this species harbors the fewest xenologous genes and relatively short “end” domains, yet it contains approximately 35-fold more transposable elements than *W. versatilis*. These findings indicate that transposon abundance is not positively associated with xenologous gene content, although we cannot presently exclude the possibility of a negative correlation between the two. A hypothetical negative correlation might be explained by a contribution of TEs to xenologous gene loss and genomic reorganization by providing homologous sequences scattered throughout the genome that facilitate recombination^63^. Transposable element content also seems to be unrelated with the length of “end” domains and the extent of transcriptional repression found therein. Although proximity to TEs was found to negatively impact expression, as previously reported^63^ and TEs are found mainly in “end” domains, the number of impacted genes is too low to explain the overall depression of transcription in “end” domains. Instead, the proximity of TEs may add an additional layer of transcriptional repression to a fraction of the genes in “end domains”, possibly by eliciting the local formation of additional repressive chromatin structures^63^. Finally, although the elucidation of the mechanisms by which foreign DNA was incorporated by W/S-clade genomes is outside the scope of the present work, we note that a prominent TE vector for HGT in the *Pezizomycotina*, the starship transposons, were found to be absent from W/S clade genomes available.

In our working hypothesis, the existence of “end” domains as found in *W. versatilis* may have been an intrinsic property of chromosome architecture in the ancestor of the *Starmerella* sub-clade —or possibly, the ancestor of the entire W/S-clade. Such lengthy regions with repressed transcription at chromosome ends do not seem to be a general feature in the order Dipodascales, since they were not detected in *Y. lipolytica* and *Su. lignohabitans* and in a species phylogenetically very close to the W/S clade, *C. incommunis*. The loss of DNA-repair genes in the W/S-clade ancestor, which is more pronounced than observed in neighboring species^41^, may have favored the spread of compact chromatin towards the middle of the chromosomes, over much longer distances than observed so far in fungi^38^ and may also have caused AT-enrichment in these regions as previously proposed^59^. Still, “end” domains as reported here for W/S-clade species are not a necessary consequence of DNA repair defects, since we found that a distantly related yeast species, belonging to a lineage also defective in cell-cycle and DNA repair genes, *Hanseniaspora uvarum*, lacks this kind of “end” domain, as shown in Fig. S10O–U.

The empirical evidence here provided is consistent with the notion that “end” domains may have provided a favorable environment for successful HGT in three manners. Firstly, we observed lower gene density in “end” domains (Fig. 5), which should improve HGT success by decreasing the chance that integration of foreign genes has immediate deleterious consequences. Secondly, we found significantly higher rates of protein sequence evolution in xenologous genes located in “end” domains, when compared with xenologous genes in “middle” domains (Fig. 6). We posit that this suggests “end” domains foster adaptation of xenologous genes, because native genes that are also lowly transcribed in “end” domains do not have significantly increased d*N*/d*S* rates, when compared with native genes in “middle” domains. Thirdly, we show that “end” domains are not a common feature in the Dipodascales order; significantly, they were absent in close relatives of the W/S clade. The fact that species (*W. versatilis* and *St. bombicola*) representing the entire diversity range encompassed by the *Starmerella* sub-clade (Fig. 1) have “end” domains as defined in this work, suggests that the ancestor of at least the *Starmerella* sub-clade is likely to have possessed such regions. Since AT-enriched “end” domains have not been, to our knowledge, previously reported in yeasts, this is the most parsimonious explanation for the presence of “end” domains in two species as diverse as *St. bombicola* and *W. versatilis* within the *Starmerella* sub-clade. The alternative, which is that “end” domains evolved independently in the two species, seems unlikely, although possible. In summary, (i) the fact that no other yeast lineage exhibits as high a rate of HGT as the W/S clade^1^, that so far “end” domains were also not reported in other yeasts, (ii) that within the W/S clade, so far, lengthier “end” domains correlate with the presence of more xenologous genes and (iii) that “end” domains foster adaptation of xenologous genes through higher evolution rates, prompts us to put forward the possibility that “end” domains helped promote HGT in W/S-clade ancestors.

Another important aspect we and others^10,13^ previously addressed while studying the conversion of a bacterial operon in a eukaryotic gene cluster is the adaptation of gene expression in interdomain HGT events. Although we did not investigate if noisy (stochastic) transcription takes place in W/S clade “end” domains, in *S. cerevisiae*, transcription at subtelomeres was described to be much noisier than transcription happening in central chromosomal regions, where essential, highly expressed genes tend to be located^28^. Importantly, transcriptional noise characteristic of subtelomeric regions^28,64^ could provide an initial means of expression to xenologous genes devoid of a proper promoter upon arrival in their new host. This possibility is compatible with the idea that evolutionary processes at work would gradually relocate highly expressed xenologous genes under strong purifying selection to central regions of the chromosomes^30^ with open chromatin and low transcriptional noise, while also causing gene loss due to selection or drift. These processes seem to have advanced much further in *St. bombicola* than in *W. versatilis*. Altogether, *St. bombicola*, with its considerably lower number of xenologous genes, almost all expressed under the conditions we tested and distributed more evenly across the genome, could represent a more streamlined genome, with a larger proportion of well-adapted, more mature xenologous genes.

## Methods

### Yeast strains and growth conditions

*Wickerhamiella versatilis* PYCC 7102, *Wickerhamiella domercqiae* PYCC 3067, and S*tarmerella bombicola* PYCC 5882 were obtained from the Portuguese Yeast Culture Collection (PYCC), NOVA FCT, Caparica, Portugal. All strains were maintained in YPD medium [1% (w/v) yeast extract, 2% (w/v) peptone, 2% (w/v) glucose].

### Gene functional characterization

Functional characterization of the whole proteome and comparisons between the subsets of native and horizontally acquired protein-coding genes was performed using the PANTHER classification system^20^, following the guidelines for genomes not included in the Reference Proteome Project^65^. The full proteome was functionally classified using the PANTHER HMM scoring tool that uses the hmmsearch program against the PANTHER HMM library (library of HMMER3 models^66^), and generates a PANTHER generic mapping file. Analyses were performed with PANTHER v18.0 (released 2023-08-01), PANTHER GO-Slim (molecular function, cellular component, and biological process), and PANTHER protein class annotations. Only top-scoring HMM functional inferences with *E* < 10^−6^ were included in the analyses. EggNOG-mapper v2.1.13^21^ and InterProScan v.107.0 were further used in xenologous genes with no other functional identification. EggNOG-mapper was run in DIAMOND search mode^67^ against the EggNOG v5.0 database^68^, and as in previous PANTHER analysis, only annotations supported by hits with *E* < 10^−6^ were retained. EggNOG-mapper was also run in HMMER search mode^69^, using EggNOG v.5.0 taxon-specific databases: the bacteria database for xenologous genes inferred to originate from bacteria, and the fungal database for xenologous genes originated in *Pezizomycotina*. InterProScan was run on the web interface (https://www.ebi.ac.uk/interpro/) in default mode.

### KEGG functional enrichment analysis and pathway reconstruction

We used BlastKOALA v2.2^70^ to assigned KO (KEGG Orthology) identifiers to each gene. Xenologous genes with eucaryotic origin were submitted to a conventional KO assignment using the KEGG database genus_eukaryotes.pep, while KO classification of genes of bacterial origin resulted from the manual curation of two independent searches using the prokaryote database (genus_prokaryotes.pep) and the eukaryote database (genus_eukaryotes.pep). If these searches led to different KO identifiers, the one with the highest score was selected. The KEGG Mapper Reconstruct tool^18,19^ was used to link KO annotations to KEGG pathway maps and BRITE hierarchies using either the web platform or the biopython module Bio.KEGG.REST. We kept all retrieved KEGG categories with the exception of “organismal systems”, “human diseases”, “poorly characterized” and “without KO” that were considered not relevant for this study. Enrichment analysis in pathways of interest, namely pathways related with carbohydrate metabolism, amino acid metabolism, and metabolism of cofactors and vitamins, were performed to test if these pathways were significantly enriched in xenologous genes using Fisher's exact test with Benjamini–Hochberg correction for multiple testing.

### Prediction and annotation of transposable elements

An exhaustive analysis of the TE content in the three nanopore genome assemblies of *W. versatilis*, *W. domercqiae*, and *St. bombicola* was performed using the Earl Grey TE annotation pipeline v7.0.1^33^. The -e option was enabled to specifically identify Helitrons using HELIANO v.1.3.1^71^; all other parameters were kept at their default settings as in Leal-Dutra et al.^72^. The TE consensus library and GFF annotation file obtained for each genome assembly are provided in Supplementary Data 20. To assess the effect of TE insertions on the expression of nearby genes, these were classified into four classes based on the presence of TEs within 1 kb window upstream and/or downstream of the gene. Genes overlapping with TEs were excluded from this analysis, as were small simple repeats (microsatellites).

The presence of starship transposon elements was investigated using a combination of HMM-based protein searches and a dedicated starship detection pipeline. Protein sequences annotated as DUF3435 (captain) and DUF3723 were identified in the supplementary Table S11 of Gluck-Thaler et al.^73^ and retrieved from the corresponding fasta file deposited in FigShare (10.6084/m9.figshare.17185880); selected sequences are here provided in Supplementary Data 21. These sequences were aligned independently using MAFFT v7.525^74^. Hidden Markov model (HMM) profiles were constructed for each group using HMMER v3.4^35^ and used to assess their presence on the W/S-clade genomes with Orthofisher v.1.0.5^36^ using default parameters and an e-value cutoff of 10^−10^. In parallel, starship elements were detected using STARFISH v1.0.0^37^ under default settings.

### Identification of horizontally acquired genes

Genes originating from bacteria were previously identified using the alien index methodology^9,15,16^. Gene transfer events from *Pezizomycotina* to the W/S-clade were identified using a novel workflow that relies on the analysis of the distributions of gene-tree distances among selected phylogenetic groups (Fig. S1). Briefly, for a given gene, we infer its horizontal acquisition if it clusters within the *Pezizomycotina* clade in the gene tree. This inference is supported by observing a distribution of pairwise gene-tree distances between the focal species and *Pezizomycotina* taxa that largely overlaps with the distribution obtained from comparisons exclusively among *Pezizomycotina* taxa. This workflow initiates with a customized proteome database. Alongside eight taxa from the focal group (W/S-clade), we included five additional taxa from the Dipodascaceae/Trichomonascaceae family, 50 taxa from other families of *Saccharomycotina*, 48 taxa from the *Pezizomycotina* subphylum, and four and three taxa from Taphrinomycotina and Basidiomycota, respectively, which were used to root the tree. Species selection was guided by the genome-scale phylogeny of the kingdom fungi^75^, aiming for a balanced phylogenetic species tree that adequately represents the diversity of major clades within *Saccharomycotina* and *Pezizomycotina* (Fig. S2). For each species, we obtained their respective proteomes from NCBI, prioritizing annotations based on less fragmented assemblies. Detailed information of the taxonomy and proteome features of the strains included in this study can be found in Supplementary Data 1.

OrthoFinder^76^, ran with parameters -M msa -S diamond -A mafft -T fasttree, was used to perform phylogenetic orthology inference and orthogroup identification across all proteomes within our dataset (Supplementary Data 2–5). OrthoFinder infers rooted gene trees for each orthogroup and an overall species tree that was congruent with the phylogeny from Li et al.^75^ (Fig. S2). For each orthogroup, we quantified gene copy numbers and the relative frequency of taxa from each taxonomic group.

Orthogroups with fewer than five homologous gene copies from the *Pezizomycotina* were considered to provide insufficient basis to infer HGT from the *Pezizomycotina*. This possibly resulted in underestimation of HGT from the *Pezizomycotina* into the genome of the focal species. For orthogroups harboring more than five gene copies from the *Pezizomycotina*, frequencies were compared as follows (schematised in Fig. S1 and Supplementary Data 18):

For each orthogroup, we estimated gene-tree distance between the focal W/S-clade species and (i) all other individuals of the Dipodascaceae/Trichomonascaceae family (focal-family), and (ii) all individuals within the *Pezizomycotina* clade (focal-*Pezizomycotina*). These distances were then compared with gene-tree distances calculated within Dipodascaceae/Trichomonascaceae family, excluding the W/S-clade species (within family), and within the *Pezizomycotina* clade (within *Pezizomycotina*). To measure gene-tree distances, we used the rooted gene trees produced by OrthoFinder for each orthogroup. The ETE3 get_distance function^77^ was applied to extract branch length distances between pairs of tree terminals. The resulting lists of gene-tree distances were compared by assessing the overlap of their kernel density estimations using the R package overlapping 2.1^78^. This tool yields a distribution-free overlapping index suitable for empirical data, even in the presence of multimodality^79^. This index was computed as the proportion of the overlapping area between two densities using 1024 bins for integral computations. This statistic ranges from 0% to 100%, where 0% indicates no overlap and 100% indicates perfect overlap (i.e., identical distribution between groups). Genes acquired horizontally from *Pezizomycotina* taxa are anticipated to yield “focal-*Pezizomycotina*” and “within *Pezizomycotina*” distributions of gene-tree distances that largely overlap. Conversely, for vertically inherited genes, those distributions should not significantly overlap, and the median of “focal-*Pezizomycotina*” should exceed the medians of “focal-family”, “within family”, and “within *Pezizomycotina*” (Fig. S3). We used these expectations to devise statistics that would flag orthogroups whose distributions of gene-tree distances deviate from the expectations of native (vertically inherited) genes. While native genes typically yield gene-tree distance distributions that align closely with expectations, HGT from *Pezizomycotina* represents just one of many potential complexities in gene tree evolution that can result in divergent distributions. Therefore, we employed manual curation for orthgroups in which the focal species had multiple gene copies (multiply-copy orthogroups), as these are expected to produce more complex gene-tree distance distributions than orthogroups containing only a single gene copy from the focal species (single-copy orthogroups). In addition, all HGT inferences were validated by manual inspection of gene trees and, when justified, by conducting tblastn searches of the focal sequences against the NCBI nr database. These analyses were conducted using RStudio 2022.7.2.576^80^ with R v4.2.2^81^. Custom-made scripts, selected proteomes, and orthogroup gene trees were deposited in the Figshare repository.

### Classification of the role of horizontally acquired genes

The role/impact of horizontally acquired genes on the host genome was evaluated by determining whether their acquisition resulted in the reinforcement of a pre-existing function, replacement of a previous lost function, or in the gain of a new function (Fig. S5). Each HGT gene with a KO identifier (approximately half of the total number of genes found in each species) was examined. *Reinforcement* events were defined by the co-existence of xenologous genes and native orthologues with identical KO identifiers. The decision to classify a xenologous gene either as novel function or as a replacement was guided mainly by the detailed analysis of OrthoFinder-generated gene trees. For the least complex ones, we assumed the xenologous gene represented a new function if NO other yeast homologues were found in the orthogroup or by Blast search, which extended the survey to all publicly available yeast genomes. If this search yielded yeast homologs, the xenologous gene under study would be considered a replacement of a gene previously lost in the W/S clade lineage. Otherwise, it would be considered a new function.

In complex gene trees, this procedure was employed for each branch/sub-clade separately to assess whether all the orthogroup members were likely to fulfill the same function or instead, proteins positioned in different branches were likely to corresponded to (slightly) different functions. This was done by analyzing functionally characterized homologs retrieved from tblastn searches, thereby allowing the assignment of (slightly) different functions to distinct branches of the phylogenetic tree. When this happened, the branch harboring the xenologous gene would be examined using the same criteria employed for simple trees, i.e., the absence of yeast homologs resulted in classification as new function. Otherwise, the xenologous gene would be classified as a replacement.

### Culture conditions for RNA isolation

Yeast strains were pre-grown overnight in 50 mL Erlenmeyer flasks containing 10 mL of culture medium at 25 °C in a shaking incubator. The inoculum was diluted to OD_640 nm_ = 0.015 in 50 mL of growth medium in 250 mL flasks. Each strain was grown in triplicate in either YPD [1% (w/v) yeast extract, 2% (w/v) peptone and 2% (w/v) glucose] or YNB medium supplemented with 2% glucose, at 25 °C. Cells for RNA isolation were harvested in mid-exponential phase.

### RNA sequencing (RNA-seq)

Total RNA was extracted using the RiboPure^TM^ RNA Purification Kit (Invitrogen, Thermo Fisher), according to the manufacturer´s instructions, using approximately 3 × 10^8^ cells from an exponentially growing culture. Purified RNA was then treated with DNase I to remove contaminating chromosomal DNA. PCR amplification of the D1–D2 region of the large-subunit (28S) rRNA gene was performed using the LR6 and ITS5 primers (LR6: 5′-CGCCAGTTCTGCTTACC-3′; ITS5: 5′-GGAAGTAAAAGTCGTAACAAGG-3′) to assess the success of DNase I treatment. RNA was quantified using Qubit RNA BR Assay Kit (Thermo Fisher), and integrity was assessed by gel electrophoresis.

RNA samples were sequenced at the IGC genomic facility (Oeiras, Portugal). cDNA libraries were prepared using the Illumina Smart-Seq2 kit. Single-end sequencing was performed with an Illumina NextSeq 2000 instrument. An average of about 30 million single-end reads of 35–101 base pairs were obtained for each sample.

### RNA-seq analysis/transcriptome mapping

Quality of raw sequenced reads was accessed with FastQC v0.11.5^82^. Adapters and low-quality sequences were removed using Trimmomatic v0.39^83^ using the parameters ILLUMINACLIP:Nextera_adapters.fa:2:30:10:4:true, SLIDINGWINDOW:4:20, MINLEN:36. Clean reads were aligned to their respective reference genome (Supplementary Data 12) with GSNAP v2021-12-17 from the GMAP package^84^ with the function --novelsplicing enabled. Alignment quality was assessed with Qualimap v2.2.2^85^. Transcript quantification was performed using StringTie RNA-seq assembler v2.2.1^86^. BAM files were converted into count tables using the -e parameter to estimate gene expression levels as a function of the transcript coverage. For the two *Wickerhamiella* species, we used the NBRP/RIKEN annotations (Supplementary Data 12), while *St. bombicola* PYCC 5882 was annotated locally with AUGUSTUS v3.3.3^87^ using the *Saccharomyces cerevisiae* S288C genome as reference. AUGUSTUS gene annotation was performed in both strands (--strand = both), for complete genes (--gene model = complete), including the protein sequences (--protein = on), introns (--introns = on), coding sequences (--cds = on and --codingseq = on), start (--start = on) and stop codons (--stop = on) estimations. Average gene expression was determined for each growth media (YPD and YNB), and a gene was considered expressed when the average expression was equal or higher than 5 TPM, similarly to what was used in Shen et al.^1^.

### Differential gene expression analysis

Transcript count tables were processed using the R package EdgeR v3.36.0^88^ to determine the cohort of differential expressed genes between the two growth conditions tested. Lowly expressed genes were filtered out using the function filterByExpr, and the libraries were normalized with the function calcNormFactors that applies a trimmed mean of *M*-values (TMM) to estimate effective library sizes. A design matrix of two groups with three replicates each was created to analyse gene expression levels as a function of the growth conditions. Normalized count data were fitted to a negative binomial generalized linear model with the glmQLfit function (robust = TRUE). Differential expression was determined by applying a quasi-likelihood (QL) F test (glmQLFTest function) by defining the null hypothesis as the equality of mean expression levels between the two growth conditions. Significant results were determined for genes with FDR cut-off of 5% using the Benjamini–Hochberg method, and fold-change significantly above 1.2 as determined with the function glmTreat.

### DNA extraction and sequencing

Genomic DNA for Oxford Nanopore Technologies sequencing was extracted from overnight-grown cultures using the Quick-DNA Fungal/Bacterial Miniprep Kit (Zymo Research) following the manufacturer’s instructions. DNA purity was assessed using a NanoDrop spectrophotometer (Thermo Fisher Scientific), and DNA concentration was quantified with the Qubit dsDNA Assay Kit (Invitrogen) on a Qubit fluorometer. Oxford Nanopore whole genome sequencing was performed in-house for *Wickerhamiella versatilis* PYCC 7102, *W. domercqiae* PYCC 3067, *Starmerella bombicola* PYCC 5882, and *Candida incommunis* PYCC 4837. Libraries were prepared using the Native Barcoding Kit 24 v14 (SBK-NBD114.24) following the manufacturer’s protocol and sequenced on an R10 (FLO-MIN114) flow cell for 48–72 h at default voltage using a MinION Mk1B system and MinKNOW software v23.07.8. Guppy v6.5.7 was used for basecalling.

### Chromosome-level assemblies and genome-wide statistics

Long-read data were de novo assembled with CANU v2.2^89^ with default parameters, adjusting only the genome size (10 Mb in the case of *Starmerella*/*Wickerhamiella* and 12 MB in the case of *Candida incommunis*). The resulting contigs were polished with two rounds of Racon v1.5.0^90^, first using nanopore reads and then using Illumina reads available for the same strains (Supplementary Data 12). Assemblies were further polished with Pilon v1.24^91^ using Illumina data for multiple rounds until no further changes were reported in the Pilon change file. Scaffolding was performed with LINKS v1.8.4^92^ run interactively with *k* = 20; the distance parameter (-*d*) was increased from 1000 to 10,000 bp (stepwise), while the minimum link threshold (-*t*) was decreased from 10 to 2 to progressively improve scaffold contiguity.

De novo detection of telomeric motif sequences was performed by searching for tandem repeated sequences on all chromosome ends using Telfinder^93^, where stretches of terminal 60 kb were analyzed using a kmer window size that ranged from 5 to 60 nucleotides. These results were confirmed with tidk^94^ in “search” mode by screening the whole assembly for the presence of Telfinder repeats. Assembly completeness was further assessed with BUSCO v6.0.0^95^ using the *saccharomycetes_odb12* lineage dataset (2319 genes from 75 species; creation date 2025-07-01). Analyses were run in genome mode (*euk_genome_aug*) with Augustus v3.5.0^96^ as the gene predictor. Each assembly was evaluated on the basis of BUSCO genes classified as complete (single-copy or duplicated), fragmented or missing.

Average gene expression was mapped to chromosome-level assemblies to study how levels of expression varied along the chromosome in native and HGT genes. Mitochondrial and ribosomal RNA-only scaffolds were not included in this analysis. Gene density along the chromosomes was estimated by quantifying the total length occupied by genes in 100 kb sliding windows. Chromosome variation in base composition was estimated in two ways. Deviations from the genome average base composition was estimated in 1000-nucleotide sliding windows using seqkit^97^. Then OcculterCut^32^ was used to determine whether there was evidence for the presence of two distinct GC content region types—AT-rich (R0 region) and GC-equilibrated (R1 region). OcculterCut was initially run with default parameters to determine whether it identified a GC content cut-off that segregates the genome into distinct base composition regions. Then we fixed that cut-off value and re-run OcculterCut to classify chromosome segments.

Significant enrichment of xenologous genes in chromosomal “end” domains was analysed using two complementary approaches. First, a chi-square goodness-of-fit test was used to compare the observed number of xenologous genes identified at “end” domains with expected counts assuming random distribution of xenologous genes along the genome that was obtained by multiplying the proportion of the genome occupied by those regions to the total number of xenologous genes identified. The observed count of xenologous genes was determined directly from the annotated dataset of Supplementary Data 13. Second, a nonparametric permutation test was performed where gene labels were randomly reassigned to known gene positions, preserving the total number of xenologous genes and the same genomic structure and gene density across chromosomes. In each of 10,000 iterations, the number of xenologous genes located within “end” domains was recorded, generating a null distribution. The empirical *P*-value was calculated as the proportion of permutations yielding a count equal to or greater than that observed (one-tailed test). This approach provides a robust estimate of enrichment significance, independent of distributional assumptions. Enrichment analyses were conducted in Python v.3.11.10. Data handling was performed using pandas v2.2.3, random sampling and numerical operations with NumPy v2.2.1, and data visualization with Matplotlib v3.10.0. Chi-square tests were carried out using the *chi-square* function from scipy.stats module (SciPy v1.14.1).

### Rates of nonsynonymous and synonymous substitutions (d*N*/d*S*) and gRSCU

To assess patterns of nonsynonymous to synonymous substitution rates in genes distributed across different chromosomal regions of the *W. versatilis* genome, one-to-one orthologous sequences were identified between the proteomes of *W. versatilis* and a closely related *Wickerhamiella* sp. strain, which shared 72% nucleotide identity. Orthology inference was performed using OrthoFinder^76^, as previously described. Protein sequences were aligned with MAFFT using the E-INS-i strategy and --maxiterate 1000^74^. These alignments served as reference for codon-based alignment of nucleotide sequences in pal2nal v.14 with the -nogap option^98^.

Pairwise estimates for d*N*, d*S*, and d*N*/d*S* were computed for each gene alignment using a maximum likelihood method with an F3x4 codon model, as implemented in codeml from the PAML v.4.10.7 package (M0; runmode −2)^45^. To avoid unreliable d*N*/d*S* estimates from highly divergent sequences, alignments where d*S* exceeded 5 were excluded from all downstream analyses, following the author’s recommendations^46^. One alignment produced an outlier d*N*/d*S* value of 1.02, but excluding this data point did not alter the conclusions.

A total of 2864 alignments were kept for further analysis, corresponding to 126 and 2738 genes located at terminal and central chromosomal regions, respectively. Tests for positive selection on genes at the chromosomal termini were performed using maximum-likelihood methods and codon models as implemented in codeml (PAML v.4.10.7)^45^. Codeml was run with Nssites 0, 1, 2, 7, 8, to assess whether two d*N*/d*S* classes were necessary (M1a vs. M0 test; nearly neutral model of evolution) and to test for evidence of positive selection (M2a vs. M1a and M8 vs. M7). The significance of likelihood ratio tests was approximated to a chi-square distribution, and significant levels were Bonferroni corrected.

Gene-wise relative synonymous codon usage (gRSCU) was estimated with BioKIT v.1.1.3.^99^ using the mean synonymous codon usage value for all codons in each gene.

### Statistics and reproducibility

The Mann–Whitney *U* tests were performed in R v4.2.2 using the wilcox.test() function from the stats package. The reported Wilcoxon rank-sum statistic (W) was converted to the Mann–Whitney *U* statistic to reflect the number of times observations in one group ranked higher than those in the other. All other statistical tests are specified in the figure legends or in the “Methods” section, including the test statistic, package, and version number. Where applicable, adjustments to the significance threshold (e.g., FDR, Bonferroni) are indicated. Source data for all figures are provided in Supplementary Data 19.

### Reporting summary

Further information on research design is available in the Nature Portfolio Reporting Summary linked to this article.

## Supplementary information

Supplementary Information

Description of Additional Supplementary Files

Supplementary Data 1

Supplementary Data 2

Supplementary Data 3–5

Supplementary Data 6–8

Supplementary Data 9

Supplementary Data 10

Supplementary Data 11–12

Supplementary Data 13

Supplementary Data 14

Supplementary Data 15

Supplementary Data 16

Supplementary Data 17

Supplementary Data 18

Supplementary Data 19

Supplementary Data 20

Supplementary Data 21

Reporting Summary

## Data Availability

Numerical Source data for charts/graphs can be found in Supplementary Data 19. New sequencing reads and genome assemblies have been deposited in the Sequence Read Archive (SRA) and Genbank at the National Center for Biotechnology Information (NCBI) under accession numbers JBHGPL000000000, JBHTQU000000000, JBHTQV000000000, JBQOUZ000000000, SAMN43108609-SAMN43108626, SRR30194991-SRR30195008, SRR35017601, SRR38099987-SRR38099989, SRR38103677, and SRR38116034. Accession numbers and unique identifiers of original data, as well as data obtained from public databases, are listed in Supplementary Data 1, 11, 12, and 15. Supplementary Data 1–21, and nanopore assemblies with respective BUSCO orthologous lists have been deposit in Figshare and are accessible through the following links: 10.6084/m9.figshare.29924882^100^; 10.6084/m9.figshare.30085981^101^

## Code availability

Custom scripts have been deposit in Figshare and are accessible through the following link: 10.6084/m9.figshare.26527765^102^;

## References

[1] Shen, X.-X. et al. Tempo and mode of genome evolution in the budding yeast subphylum. *Cell* **175**, 1533–1545 (2018). 30415838 10.1016/j.cell.2018.10.023 PMC6291210

[2] Opulente, D. A. et al. Genomic factors shape carbon and nitrogen metabolic niche breadth across Saccharomycotina yeasts. *Science* **384**, eadj4503 (2024). 38662846 10.1126/science.adj4503 PMC11298794

[3] Riley, R. et al. Comparative genomics of biotechnologically important yeasts. *Proc. Natl. Acad. Sci. USA*. **113**, 9882–9887 (2016). 27535936 10.1073/pnas.1603941113 PMC5024638

[4] Krassowski, T. et al. Evolutionary instability of CUG-Leu in the genetic code of budding yeasts. *Nat. Commun.* **9**, 1887 (2018). 29760453 10.1038/s41467-018-04374-7 PMC5951914

[5] Helsen, J. et al. Gene loss predictably drives evolutionary adaptation. *Mol. Biol. Evol.* **37**, 2989–3002 (2020). 32658971 10.1093/molbev/msaa172 PMC7530610

[6] Marsit, S., Sanchez, I., Galeote, V. & Dequin, S. Horizontally acquired oligopeptide transporters favour adaptation of *Saccharomyces cerevisiae* wine yeast to oenological environment. *Environ. Microbiol.* **18**, 1148–1161 (2016). 26549518 10.1111/1462-2920.13117

[7] Eberlein, C., Abou Saada, O., Friedrich, A., Albertin, W. & Schacherer, J. Different trajectories of polyploidization shape the genomic landscape of the *Brettanomyces bruxellensis* yeast species. *Genome Res.* **31**, 2316–2326 (2021). 34815309 10.1101/gr.275380.121 PMC8647821

[8] Gonçalves, P., Gonçalves, C., Brito, P. H. & Sampaio, J. P. The *Wickerhamiella/Starmerella* clade—a treasure trove for the study of the evolution of yeast metabolism. *Yeast* **37**, 313–320 (2020). 32061177 10.1002/yea.3463

[9] Gonçalves, C. et al. Evidence for loss and reacquisition of alcoholic fermentation in a fructophilic yeast lineage. *eLife* **7**, e33034 (2018). 29648535 10.7554/eLife.33034 PMC5897096

[10] Gonçalves, C. & Gonçalves, P. Multilayered horizontal operon transfers from bacteria reconstruct a thiamine salvage pathway in yeasts. *Proc. Natl. Acad. Sci. USA*. **116**, 22219–22228 (2019). 31611373 10.1073/pnas.1909844116 PMC6825263

[11] Gonçalves, C., Coelho, M. A., Salema-Oom, M. & Gonçalves, P. Stepwise functional evolution in a fungal sugar transporter family. *Mol. Biol. Evol.* **33**, 352–366 (2016). 26474848 10.1093/molbev/msv220

[12] Sun, L. et al. Functional and evolutionary integration of a fungal gene with a bacterial operon. *Mol. Biol. Evol.* **41**, msae045 (2024). 38415839 10.1093/molbev/msae045 PMC11043216

[13] Kominek, J. et al. Eukaryotic acquisition of a bacterial operon. *Cell* **176**, 1356–1366 (2019). 30799038 10.1016/j.cell.2019.01.034 PMC7295392

[14] Pontes, A. et al. Extensive remodeling of sugar metabolism through gene loss and horizontal gene transfer in a eukaryotic lineage. *BMC Biol.* **22**, 128 (2024). 38816863 10.1186/s12915-024-01929-7 PMC11140947

[15] Alexander, W. G., Wisecaver, J. H., Rokas, A. & Hittinger, C. T. Horizontally acquired genes in early-diverging pathogenic fungi enable the use of host nucleosides and nucleotides. *Proc. Natl. Acad. Sci. USA*. **113**, 4116–4121 (2016). 27035945 10.1073/pnas.1517242113 PMC4839431

[16] Gladyshev, E. A., Meselson, M. & Arkhipova, I. R. Massive horizontal gene transfer in Bdelloid rotifers. *Science* **320**, 1210–1213 (2008). 18511688 10.1126/science.1156407

[17] Kanehisa, M., Sato, Y., Kawashima, M., Furumichi, M. & Tanabe, M. KEGG as a reference resource for gene and protein annotation. *Nucleic Acids Res.* **44**, D457–D462 (2016). 26476454 10.1093/nar/gkv1070 PMC4702792

[18] Kanehisa, M. & Sato, Y. KEGG mapper for inferring cellular functions from protein sequences. *Protein Sci.* **29**, 28–35 (2020). 31423653 10.1002/pro.3711 PMC6933857

[19] Kanehisa, M., Sato, Y. & Kawashima, M. KEGG mapping tools for uncovering hidden features in biological data. *Protein Sci.* **31**, 47–53 (2022). 34423492 10.1002/pro.4172 PMC8740838

[20] Mi, H. et al. Protocol update for large-scale genome and gene function analysis with the PANTHER classification system (v.14.0). *Nat. Protoc.* **14**, 703–721 (2019). 30804569 10.1038/s41596-019-0128-8 PMC6519457

[21] Cantalapiedra, C. P., Hernández-Plaza, A., Letunic, I., Bork, P. & Huerta-Cepas, J. eggNOG-mapper v2: functional annotation, orthology assignments, and domain prediction at the metagenomic scale. *Mol. Biol. Evol.* **38**, 5825–5829 (2021). 34597405 10.1093/molbev/msab293 PMC8662613

[22] Blum, M. et al. InterPro: the protein sequence classification resource in 2025. *Nucleic Acids Res.* **53**, D444–D456 (2025). 39565202 10.1093/nar/gkae1082 PMC11701551

[23] Jones, P. et al. InterProScan 5: genome-scale protein function classification. *Bioinformatics* **30**, 1236–1240 (2014). 24451626 10.1093/bioinformatics/btu031 PMC3998142

[24] Rossoni, A. W. et al. The genomes of polyextremophilic cyanidiales contain 1% horizontally transferred genes with diverse adaptive functions. *eLife* **8**, e45017 (2019). 31149898 10.7554/eLife.45017 PMC6629376

[25] Smith, M. T., de Hoog, G. S., Malloch, D. & Kurtzman, C. P. *Wickerhamiella* van der Walt (1973). In *The Yeasts: A Taxonomic Study,* (eds Kurtzman, C. P., Fell, J. W. & Boekhout, T.) 891–898 (Elsevier, 2011).

[26] Gierman, H. J. et al. Domain-wide regulation of gene expression in the human genome. *Genome Res.* **17**, 1286–1295 (2007). 17693573 10.1101/gr.6276007 PMC1950897

[27] Yue, J.-X. et al. Contrasting evolutionary genome dynamics between domesticated and wild yeasts. *Nat. Genet.* **49**, 913–924 (2017). 28416820 10.1038/ng.3847 PMC5446901

[28] Batada, N. N. & Hurst, L. D. Evolution of chromosome organization driven by selection for reduced gene expression noise. *Nat. Genet.* **39**, 945–949 (2007). 17660811 10.1038/ng2071

[29] Dunn, M. J., Shazib, S. U. A., Simonton, E., Slot, J. C. & Anderson, M. Z. Architectural groups of a subtelomeric gene family evolve along distinct paths in *Candida albicans*. *G3 Genes Genomes Genet.* **12**, jkac283 (2022). 10.1093/g3journal/jkac283 PMC9713401 36269198

[30] Husnik, F. & McCutcheon, J. P. Functional horizontal gene transfer from bacteria to eukaryotes. *Nat. Rev. Microbiol.* **16**, 67–79 (2018). 29176581 10.1038/nrmicro.2017.137

[31] Wyrick, J. J. et al. Chromosomal landscape of nucleosome-dependent gene expression and silencing in yeast. *Nature* **402**, 418–421 (1999). 10586882 10.1038/46567

[32] Testa, A. C., Oliver, R. P. & Hane, J. K. OcculterCut: a comprehensive survey of AT-rich regions in fungal genomes. *Genome Biol. Evol.* **8**, 2044–2064 (2016). 27289099 10.1093/gbe/evw121 PMC4943192

[33] Baril, T., Galbraith, J. & Hayward, A. Earl Grey: a fully automated user-friendly transposable element annotation and analysis pipeline. *Mol. Biol. Evol.* **41**, msae068 (2024). 38577785 10.1093/molbev/msae068 PMC11003543

[34] Wells, J. N. & Feschotte, C. A field guide to eukaryotic transposable elements. *Annu. Rev. Genet.* **54**, 539–561 (2020). 32955944 10.1146/annurev-genet-040620-022145 PMC8293684

[35] Finn, R. D., Clements, J. & Eddy, S. R. HMMER web server: interactive sequence similarity searching. *Nucleic Acids Res.* **39**, W29–W37 (2011). 21593126 10.1093/nar/gkr367 PMC3125773

[36] Steenwyk, J. L. & Rokas, A. orthofisher: a broadly applicable tool for automated gene identification and retrieval. *G3 Genes Genomes Genet.* **11**, jkab250 (2021). 10.1093/g3journal/jkab250 PMC8496211 34544141

[37] Gluck-Thaler, E. & Vogan, A. A. Systematic identification of cargo-mobilizing genetic elements reveals new dimensions of eukaryotic diversity. *Nucleic Acids Res.* **52**, 5496–5513 (2024). 38686785 10.1093/nar/gkae327 PMC11162782

[38] Yadav, R. K., Matsuda, A., Lowe, B. R., Hiraoka, Y. & Partridge, J. F. Subtelomeric chromatin in the fission yeast *S. pombe*. *Microorganisms* **9**, 1977 (2021). 34576871 10.3390/microorganisms9091977 PMC8466458

[39] Luttermann, T. et al. Establishment of a near-contiguous genome sequence of the citric acid producing yeast *Yarrowia lipolytica* DSM 3286 with resolution of rDNA clusters and telomeres. *NAR Genom. Bioinform.* **3**, lqab085 (2021). 34661101 10.1093/nargab/lqab085 PMC8515841

[40] Bellasio, M. et al. Complete genome sequence and transcriptome regulation of the pentose utilizing yeast *Sugiyamaella lignohabitans*. *FEMS Yeast Res.* **16**, fow037 (2016). 27189363 10.1093/femsyr/fow037

[41] Gonçalves, C. et al. Stable hypermutators revealed by the genomic landscape of genes involved in genome stability among yeast species. *Mol. Biol. Evol.* **42**, msaf285 (2025). 41178256 10.1093/molbev/msaf285 PMC12629084

[42] Steenwyk, J. L. et al. Extensive loss of cell-cycle and DNA repair genes in an ancient lineage of bipolar budding yeasts. *PLoS Biol.* **17**, e3000255 (2019). 31112549 10.1371/journal.pbio.3000255 PMC6528967

[43] Pontremoli, C. et al. Evolutionary rates of mammalian telomere-stability genes correlate with karyotype features and female germline expression. *Nucleic Acids Res.* **46**, 7153–7168 (2018). 29893967 10.1093/nar/gky494 PMC6101625

[44] Anderson, M. Z., Wigen, L. J., Burrack, L. S. & Berman, J. Real-time evolution of a subtelomeric gene family in *Candida albicans*. *Genetics* **200**, 907–919 (2015). 25956943 10.1534/genetics.115.177451 PMC4512551

[45] Yang, Z. PAML 4: phylogenetic analysis by maximum likelihood. *Mol. Biol. Evol.* **24**, 1586–1591 (2007). 17483113 10.1093/molbev/msm088

[46] Yang, Z. Neutral and adaptive protein evolution. in *Computational Molecular Evolution* Ch. 8, 8259–292 (Oxford University Press, 2006).

[47] Keeling, P. J. Horizontal gene transfer in eukaryotes: aligning theory with data. *Nat. Rev. Genet.* **25**, 416–430 (2024). 38263430 10.1038/s41576-023-00688-5

[48] Liu, F., Wang, S.-H., Cheewangkoon, R. & Zhao, R.-L. Uneven distribution of prokaryote-derived horizontal gene transfer in fungi: a lifestyle-dependent phenomenon. *mBio* **16**, e02855–24 (2025). 39611838 10.1128/mbio.02855-24 PMC11708051

[49] De Paula, G. T., Menezes, C., Pupo, M. T. & Rosa, C. A. Stingless bees and microbial interactions. *Curr. Opin. Insect Sci.* **44**, 41–47 (2021). 33271364 10.1016/j.cois.2020.11.006

[50] Richards, T. A., Leonard, G., Soanes, D. M. & Talbot, N. J. Gene transfer into the fungi. *Fungal Biol. Rev.* **25**, 98–110 (2011).

[51] Moreira, D. & López-García, P. Protist evolution: stealing genes to gut it out. *Curr. Biol.* **27**, R223–R225 (2017). 28324738 10.1016/j.cub.2017.02.010 PMC5650046

[52] Van Etten, J. & Bhattacharya, D. Horizontal gene transfer in eukaryotes: not if, but how much? *Trends Genet.* **36**, 915–925 (2020). 33012528 10.1016/j.tig.2020.08.006

[53] Nosenko, T. & Bhattacharya, D. Horizontal gene transfer in chromalveolates. *BMC Evol. Biol.* **7**, 173 (2007). 17894863 10.1186/1471-2148-7-173 PMC2064935

[54] Metcalf, J. A., Funkhouser-Jones, L. J., Brileya, K., Reysenbach, A.-L. & Bordenstein, S. R. Antibacterial gene transfer across the tree of life. *eLife* **3**, e04266 (2014). 25422936 10.7554/eLife.04266 PMC4241558

[55] Katz, L. A. Recent events dominate interdomain lateral gene transfers between prokaryotes and eukaryotes and, with the exception of endosymbiotic gene transfers, few ancient transfer events persist. *Philos. Trans. R. Soc. B* **370**, 20140324 (2015). 10.1098/rstb.2014.0324 PMC4571564 26323756

[56] Brown, C. A., Murray, A. W. & Verstrepen, K. J. Rapid expansion and functional divergence of subtelomeric gene families in yeasts. *Curr. Biol.* **20**, 895–903 (2010). 20471265 10.1016/j.cub.2010.04.027 PMC2877759

[57] Louis, E. J. & Haber, J. E. The structure and evolution of subtelomeric Y’ repeats in *Saccharomyces cerevisiae*. *Genetics* **131**, 559–574 (1992). 1628806 10.1093/genetics/131.3.559 PMC1205030

[58] Kitada, T. et al. Mechanism for epigenetic variegation of gene expression at yeast telomeric heterochromatin. *Genes Dev.* **26**, 2443–2455 (2012). 23124068 10.1101/gad.201095.112 PMC3490002

[59] Katju, V. & Bergthorsson, U. Old trade, new tricks: insights into the spontaneous mutation process from the partnering of classical mutation accumulation experiments with high-throughput genomic approaches. *Genome Biol. Evol.* **11**, 136–165 (2019). 30476040 10.1093/gbe/evy252 PMC6330053

[60] Dabin, J., Mori, M. & Polo, S. E. The DNA damage response in the chromatin context: a coordinated process. *Curr. Opin. Cell Biol.* **82**, 102176 (2023). 37301060 10.1016/j.ceb.2023.102176

[61] Baltrus, D. A. Exploring the costs of horizontal gene transfer. *Trends Ecol. Evol.* **28**, 489–495 (2013). 23706556 10.1016/j.tree.2013.04.002

[62] Drummond, D. A., Bloom, J. D., Adami, C., Wilke, C. O. & Arnold, F. H. Why highly expressed proteins evolve slowly. *Proc. Natl. Acad. Sci. USA*. **102**, 14338–14343 (2005). 16176987 10.1073/pnas.0504070102 PMC1242296

[63] Castanera, R. et al. Transposable elements versus the fungal genome: impact on whole-genome architecture and transcriptional profiles. *PLoS Genet.* **12**, e1006108 (2016). 27294409 10.1371/journal.pgen.1006108 PMC4905642

[64] Hurst, L. D. Evolutionary genomics and the reach of selection. *J. Biol.* **8**, 12 (2009). 19291255 10.1186/jbiol113 PMC2687768

[65] Mi, H., Muruganujan, A., Ebert, D., Huang, X. & Thomas, P. D. PANTHER version 14: more genomes, a new PANTHER GO-slim and improvements in enrichment analysis tools. *Nucleic Acids Res.* **47**, D419–D426 (2019). 30407594 10.1093/nar/gky1038 PMC6323939

[66] Eddy, S. R. A new generation of homology search tools based on probabilistic inference. *Genome Inform.* **23**, 205–211 (2009). 20180275

[67] Buchfink, B., Reuter, K. & Drost, H.-G. Sensitive protein alignments at tree-of-life scale using DIAMOND. *Nat. Methods* **18**, 366–368 (2021). 33828273 10.1038/s41592-021-01101-x PMC8026399

[68] Huerta-Cepas, J. et al. eggNOG 5.0: a hierarchical, functionally and phylogenetically annotated orthology resource based on 5090 organisms and 2502 viruses. *Nucleic Acids Res.* **47**, D309–D314 (2019). 30418610 10.1093/nar/gky1085 PMC6324079

[69] Eddy, S. R. Accelerated profile HMM searches. *PLoS Comput. Biol.* **7**, e1002195 (2011). 22039361 10.1371/journal.pcbi.1002195 PMC3197634

[70] Kanehisa, M., Sato, Y. & Morishima, K. BlastKOALA and GhostKOALA: KEGG tools for functional characterization of genome and metagenome sequences. *J. Mol. Biol.* **428**, 726–731 (2016). 26585406 10.1016/j.jmb.2015.11.006

[71] Li, Z., Gilbert, C., Peng, H. & Pollet, N. Discovery of numerous novel *Helitron*-like elements in eukaryote genomes using HELIANO. *Nucleic Acids Res.* **52**, e79–e79 (2024). 39119924 10.1093/nar/gkae679 PMC11417382

[72] Leal-Dutra, C. A. et al. Genomic signatures of domestication in a fungus obligately farmed by leafcutter ants. *Mol. Biol. Evol.* **41**, msae197 (2024). 39288321 10.1093/molbev/msae197 PMC11451569

[73] Gluck-Thaler, E. et al. Giant starship elements mobilize accessory genes in fungal genomes. *Mol. Biol. Evol.* **39**, msac109 (2022). 35588244 10.1093/molbev/msac109 PMC9156397

[74] Katoh, K. & Standley, D. M. MAFFT multiple sequence alignment software version 7: improvements in performance and usability. *Mol. Biol. Evol.* **30**, 772–780 (2013). 23329690 10.1093/molbev/mst010 PMC3603318

[75] Li, Y. et al. A genome-scale phylogeny of the kingdom fungi. *Curr. Biol.* **31**, 1653–1665.e5 (2021). 33607033 10.1016/j.cub.2021.01.074 PMC8347878

[76] Emms, D. M. & Kelly, S. OrthoFinder: phylogenetic orthology inference for comparative genomics. *Genome Biol.* **20**, 238 (2019). 31727128 10.1186/s13059-019-1832-y PMC6857279

[77] Huerta-Cepas, J., Serra, F. & Bork, P. ETE 3: reconstruction, analysis, and visualization of phylogenomic data. *Mol. Biol. Evol.* **33**, 1635–1638 (2016). 26921390 10.1093/molbev/msw046 PMC4868116

[78] Pastore, M., Loro, P., Mingione, M. & Calcagnì, A. Overlapping: Estimation of Overlapping in Empirical Distributions. R package v2.1. https://CRAN.R-project.org/package=overlapping. (2022).

[79] Pastore, M. & Calcagnì, A. Measuring distribution similarities between samples: a distribution-free overlapping index. *Front. Psychol.* **10**, 1089 (2019). 31231264 10.3389/fpsyg.2019.01089 PMC6558420

[80] RStudio Team. RStudio: Integrated Development Environment for R. RStudio, PBC, http://www.rstudio.com/. (2022).

[81] R Core Team. R: A language and environment for statistical computing. R Foundation for Statistical Computing, https://www.R-project.org/. (2022).

[82] Andrews, S. FastQC: a quality control tool for high throughput sequence data, http://www.bioinformatics.babraham.ac.uk/projects/fastqc. (2010).

[83] Bolger, A. M., Lohse, M. & Usadel, B. Trimmomatic: a flexible trimmer for Illumina sequence data. *Bioinformatics* **30**, 2114–2120 (2014). 24695404 10.1093/bioinformatics/btu170 PMC4103590

[84] Wu, T. D. & Watanabe, C. K. GMAP: a genomic mapping and alignment program for mRNA and EST sequences. *Bioinformatics* **21**, 1859–1875 (2005). 15728110 10.1093/bioinformatics/bti310

[85] Okonechnikov, K., Conesa, A. & García-Alcalde, F. Qualimap 2: advanced multi-sample quality control for high-throughput sequencing data. *Bioinformatics* **32**, 292–294 (2016). 26428292 10.1093/bioinformatics/btv566 PMC4708105

[86] Pertea, M. et al. StringTie enables improved reconstruction of a transcriptome from RNA-seq reads. *Nat. Biotechnol.* **33**, 290–295 (2015). 25690850 10.1038/nbt.3122 PMC4643835

[87] Stanke, M., Steinkamp, R., Waack, S. & Morgenstern, B. AUGUSTUS: a web server for gene finding in eukaryotes. *Nucleic Acids Res.* **32**, W309–W312 (2004). 15215400 10.1093/nar/gkh379 PMC441517

[88] Chen, Y., Lun, A. T. L. & Smyth, G. K. From reads to genes to pathways: differential expression analysis of RNA-Seq experiments using Rsubread and the edgeR quasi-likelihood pipeline. *F1000 Res.* **5**, 1–27 (2016). 10.12688/f1000research.8987.1 PMC4934518 27508061

[89] Koren, S. et al. Canu: scalable and accurate long-read assembly via adaptive *k*-mer weighting and repeat separation. *Genome Res.* **27**, 722–736 (2017). 28298431 10.1101/gr.215087.116 PMC5411767

[90] Vaser, R., Sović, I., Nagarajan, N. & Šikić, M. Fast and accurate de novo genome assembly from long uncorrected reads. *Genome Res.* **27**, 737–746 (2017). 28100585 10.1101/gr.214270.116 PMC5411768

[91] Walker, B. J. et al. Pilon: an integrated tool for comprehensive microbial variant detection and genome assembly improvement. *PLoS ONE* **9**, e112963 (2014). 25409509 10.1371/journal.pone.0112963 PMC4237348

[92] Warren, R. L. et al. LINKS: scalable, alignment-free scaffolding of draft genomes with long reads. *GigaScience* **4**, 35 (2015). 26244089 10.1186/s13742-015-0076-3 PMC4524009

[93] Sun, Q., Wang, H., Tao, S. & Xi, X. Large-scale detection of telomeric motif sequences in genomic data using TelFinder. *Microbiol. Spectr.* **11**, e03928–22 (2023). 36847562 10.1128/spectrum.03928-22 PMC10100673

[94] Brown, M. R., Gonzalez de La Rosa, P. & Blaxter, M. tidk: a toolkit to rapidly identify telomeric repeats from genomic datasets. *Bioinformatics* **41**, btaf049 (2025). 10.1093/bioinformatics/btaf049 PMC11814493 39891350

[95] Tegenfeldt, F. et al. OrthoDB and BUSCO update: annotation of orthologs with wider sampling of genomes. *Nucleic Acids Res.* **53**, D516–D522 (2025). 39535043 10.1093/nar/gkae987 PMC11701741

[96] Stanke, M., Diekhans, M., Baertsch, R. & Haussler, D. Using native and syntenically mapped cDNA alignments to improve de novo gene finding. *Bioinformatics* **24**, 637–644 (2008). 18218656 10.1093/bioinformatics/btn013

[97] Shen, W., Sipos, B. & Zhao, L. SeqKit2: a swiss army knife for sequence and alignment processing. *iMeta* **3**, e191 (2024). 38898985 10.1002/imt2.191 PMC11183193

[98] Suyama, M., Torrents, D. & Bork, P. PAL2NAL: robust conversion of protein sequence alignments into the corresponding codon alignments. *Nucleic Acids Res.* **34**, W609–W612 (2006). 16845082 10.1093/nar/gkl315 PMC1538804

[99] Steenwyk, J. L. et al. BioKIT: a versatile toolkit for processing and analyzing diverse types of sequence data. *Genetics* **221**, iyac079 (2022). 35536198 10.1093/genetics/iyac079 PMC9252278

[100] Brito, P. et al. Regions of low gene expression promote maintenance and adaptation of horizontally acquired genes in yeasts. Communications Biology–Supplementary tables; 10.6084/m9.figshare.29924882 (2026). 10.1038/s42003-026-10153-8 PMC13376923 42103887

[101] Brito, P. et al. Regions of low gene expression promote maintenance and adaptation of horizontally acquired genes in yeasts. Communications Biology–NCBI submissions of Nanopore assemblies and BUSCO ortholog lists; 10.6084/m9.figshare.30085981 (2026). 10.1038/s42003-026-10153-8 PMC13376923 42103887

[102] Brito, P. et al. Regions of low gene expression promote maintenance and adaptation of horizontally acquired genes in yeasts. Communications Biology–Custom-made scripts; 10.6084/m9.figshare.26527765 (2026). 10.1038/s42003-026-10153-8 PMC13376923 42103887

