# Supplementary material for: Regions of low gene expression promote maintenance and adaptation of horizontally acquired genes in yeasts
Source: Commun Biol. 2026 May 9;9:968. doi: 10.1038/s42003-026-10153-8 (PMC13376923; doi:10.1038/s42003-026-10153-8)
Supplement: Supplementary file 1 — Supplementary Information [file 42003_2026_10153_MOESM1_ESM.pdf]

Supporting information for

Regions of low gene expression promote maintenance and adaptation of horizontally acquired genes in yeasts

Patrícia H. Brito[1,2*], Victoria Gil[1,2], Ana Pontes[1,2], Margarida Silva[1,2], Carla Gonçalves[1,2] and Paula Gonçalves[1,2*]

* Corresponding authors
Email: phbrito@fct.unl.pt Email: pmz@fct.unl.pt

[1] Associate Laboratory i4HB - Institute for Health and Bioeconomy, NOVA School of Science and Technology, Universidade NOVA de Lisboa, 2829-516 Caparica, Portugal

[2] UCIBIO - Applied Molecular Biosciences Unit, Department of Life Sciences, NOVA School of Science and Technology, Universidade NOVA de Lisboa, 2829-516 Caparica, Portugal

This PDF file includes:

- Supplementary Figures S1 to S13

Other supporting materials for this manuscript include custom-made scripts, supplementary tables, Nanopore assemblies and BUSCO ortholog lists that were deposited at Fishare repository:

- Supplementary tables: https://doi.org/10.6084/m9.figshare.29924882
- Custom-made scripts: https://doi.org/10.6084/m9.figshare.26527765
- NCBI submissions of Nanopore assemblies and BUSCO ortholog lists: https://doi.org/10.6084/m9.figshare.30085981

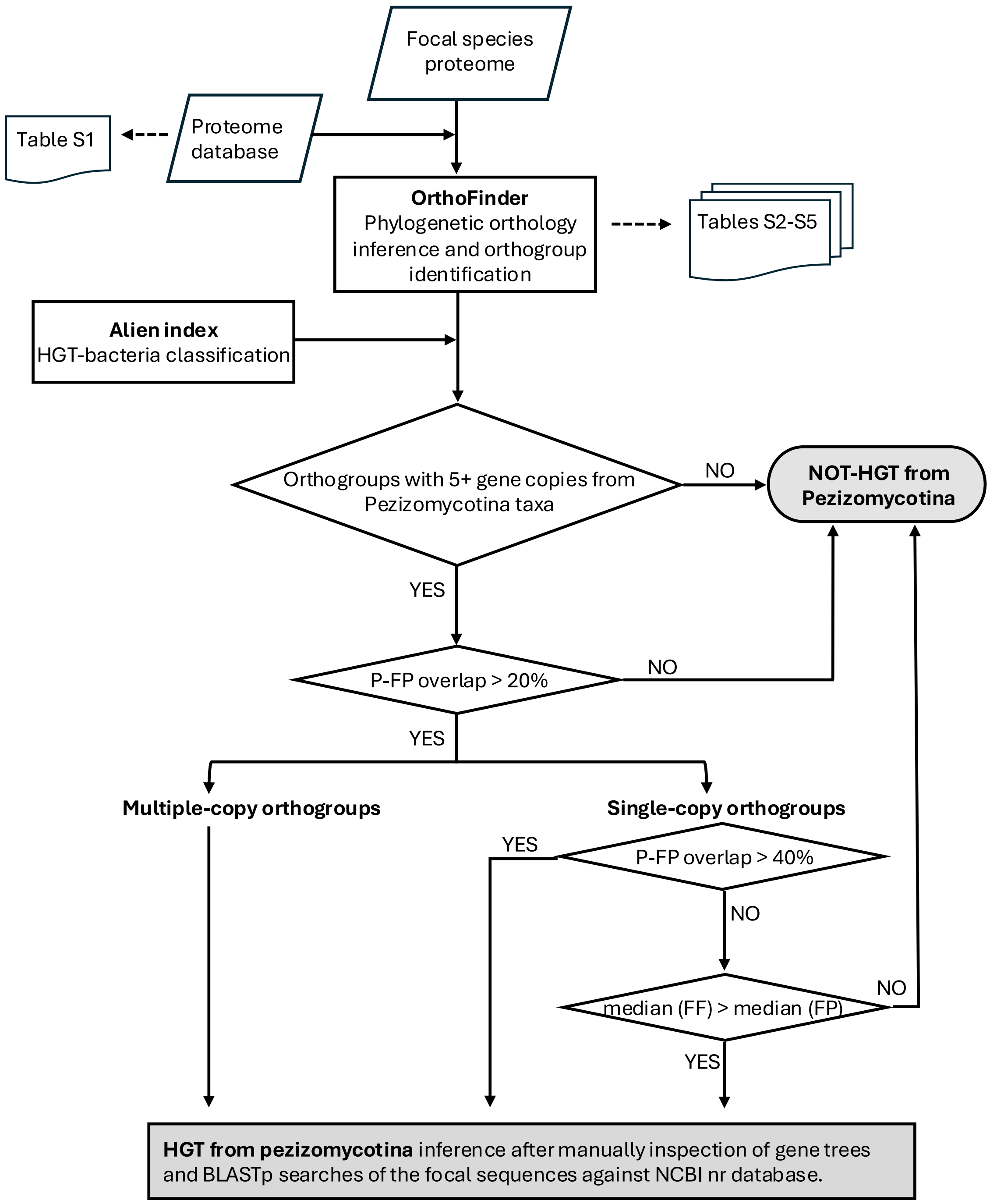

Fig. S1. **Inference of horizontally acquired genes from Pezizomycotina to the W/S clade**. Flowchart illustrating the workflow used to identify horizontally acquired genes from Pezizomycotina in W/S-clade species. The process began with OrthoFinder analysis using the proteomes of the focal W/S-clade species and a curated database containing representative species from major clades within Saccharomycotina, Pezizomycotina, and other key fungal lineages. Orthogroups with too few Pezizomycotina representatives were considered inappropriate for inference of HGT from the Pezizomycotina into the W/S clade. For the remaining orthogroups, rooted gene-trees generated by OrthoFinder were used to estimate gene-tree distance distributions, which were analyzed using overlap and median-based statistics as follows: **Focal-Family (FF)** - gene-tree distances between the focal W/S-clade

species and other members of the Dipodascaceae/Trichomonascaceae family. **Focal-Pezizomycotina (FP)** - gene-tree distances between the focal species and members of the Pezizomycotina clade. **Within-Family (F)** - gene-tree distances among Dipodascaceae/Trichomonascaceae family, excluding W/S-clade taxa. **Within-Pezizomycotina (P)** - gene-tree distances among Pezizomycotina taxa. Additionally, **P-FP overlap** quantifies the degree of overlap between P and FP distributions, while **median (FF)** and **median (FP)** refer to the median values of the FF and FP distributions, respectively. Native, vertically inherited genes generally yield gene-tree distance distributions matching expectations, whereas HGT from Pezizomycotina is one of several factors that can produce divergent distributions. Multi-copy orthogroups (orthogroups in which the focal species had multiple gene copies) underwent additional manual curation, as they are expected to yield more complex distance patterns than single-copy orthogroups (orthogroups containing only a single gene copy from the focal species). All HGT inferences were validated by manual inspection of gene trees and, when appropriate, by BLASTp searches of focal sequences against the NCBI nr database. Therefore, all these cases were manually inspected.

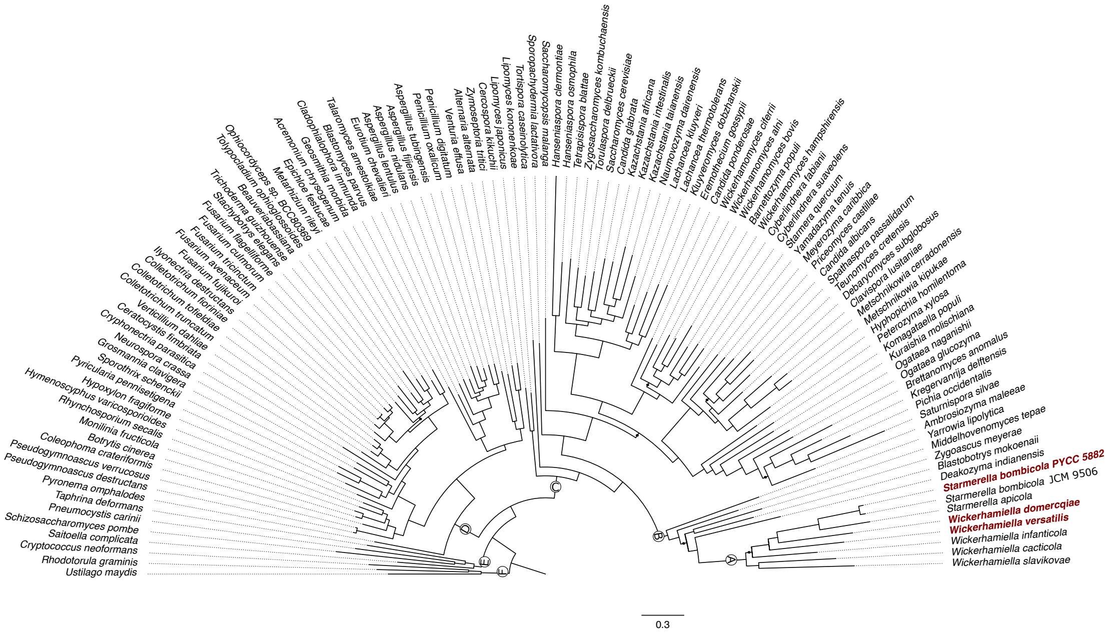

Fig. S2. Phylogeny of the fungal taxa used in the Pezizomycotina HGT pipeline. Species tree inferred using OrthoFinder with 25649 orthogroups. Tree branch lengths are measured in units of substitutions per site. Branch support values are all 100%, except for the seven internal branches marked with black circles that were not robustly recovered (less than 90% support). The three focal species analyzed in this study are highlighted in red. Letters inside circles denote main phylogenetic clades as follows: A is the W/S clade, B is the Dipodascaceae/Trichomonascaceae family, C is the Saccharomycotina subphylum, D is the Pezizomycotina subphylum, E is the Taphrinomycotina subphylum, and F is the Basidiomycota phylum.

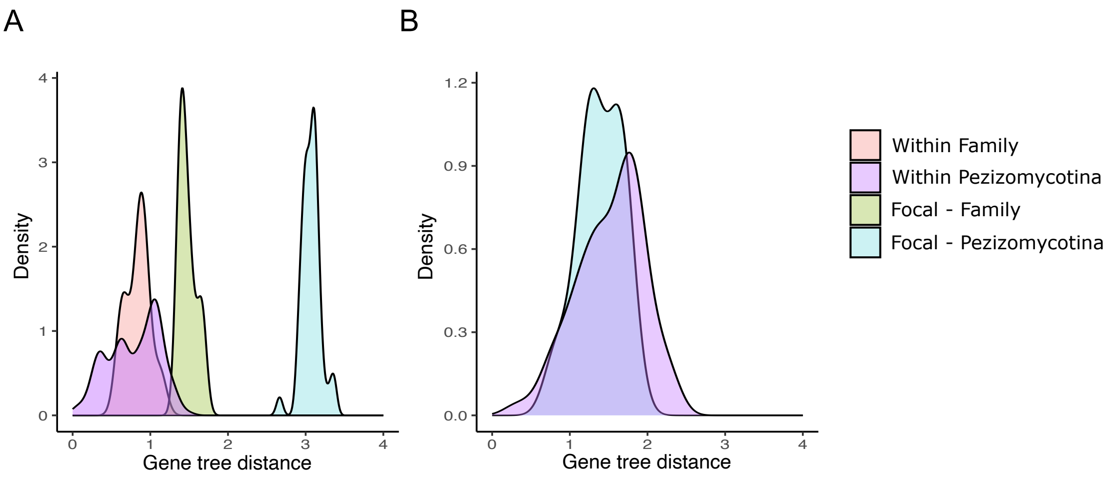

Fig. S3. Distributions of gene-tree distances. Example distributions of gene-tree distance estimated within Dipodascaceae/Trichomonascaceae family excluding the W/S species (within Family), within the Pezizomycotina clade (within Pezizomycotina), between the focal species and all other individuals of the Dipodascaceae/Trichomonascaceae family (Focal-Family), and between the focal species and all individuals within Pezizomycotina clade (Focal-Pezizomycotina). (A) represents the distribution of gene-tree distance obtained for a native, vertically inherited, gene. (B) represents the distribution of gene-tree distance obtained for a horizontally acquired gene from Pezizomycotina taxa.

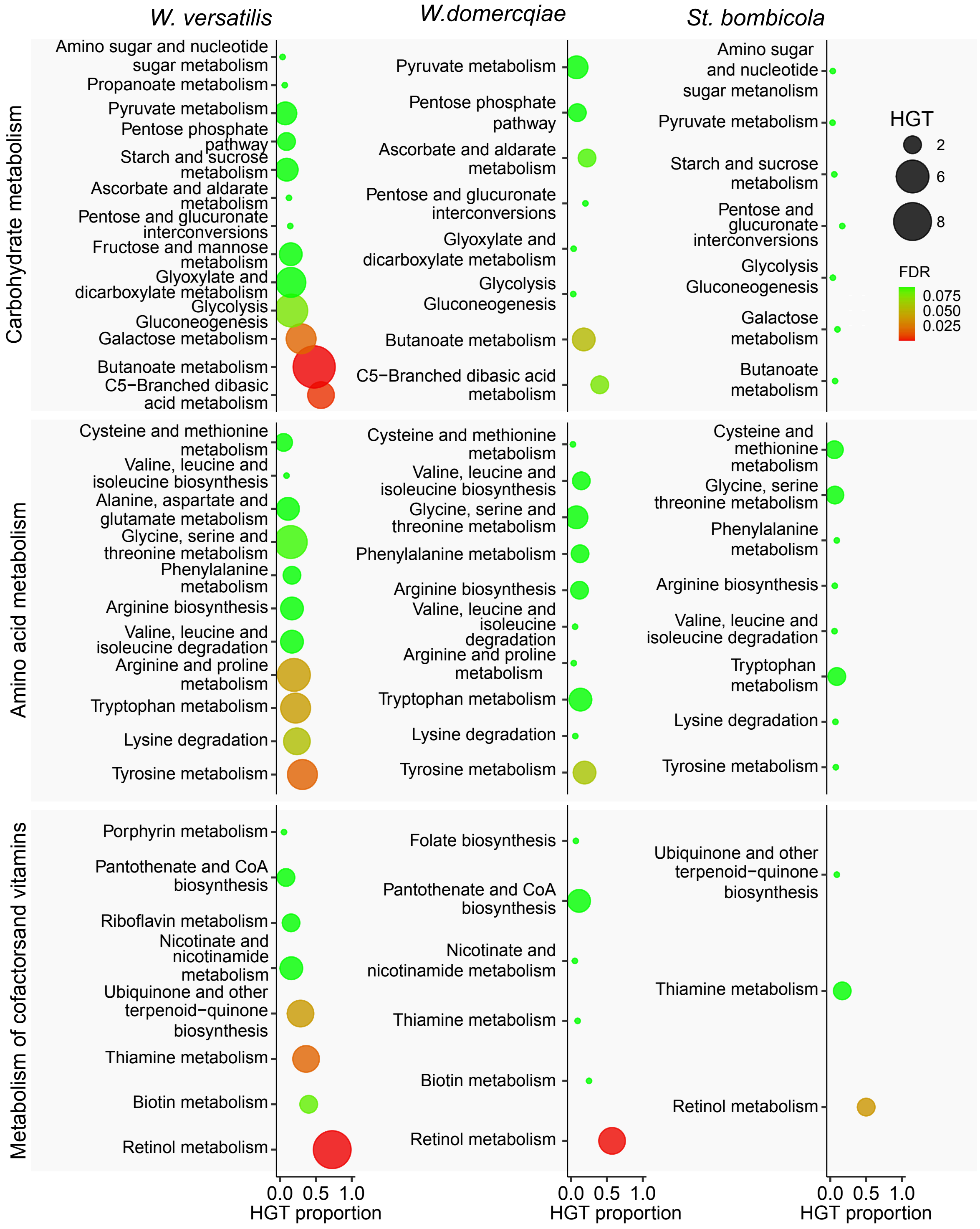

Fig. S4. Functional enrichment of horizontally acquired genes. Functional enrichment analysis was performed with KEGG pathways belonging to the metabolism category, grouped into carbohydrate, amino acid and cofactor and vitamin metabolism. Circle size indicates the total number of horizontally acquired genes in each pathway while the X-axis indicates the proportion of xenologous genes in the respective pathway. Colour indicates the corrected p-value (FDR), as depicted in the key.

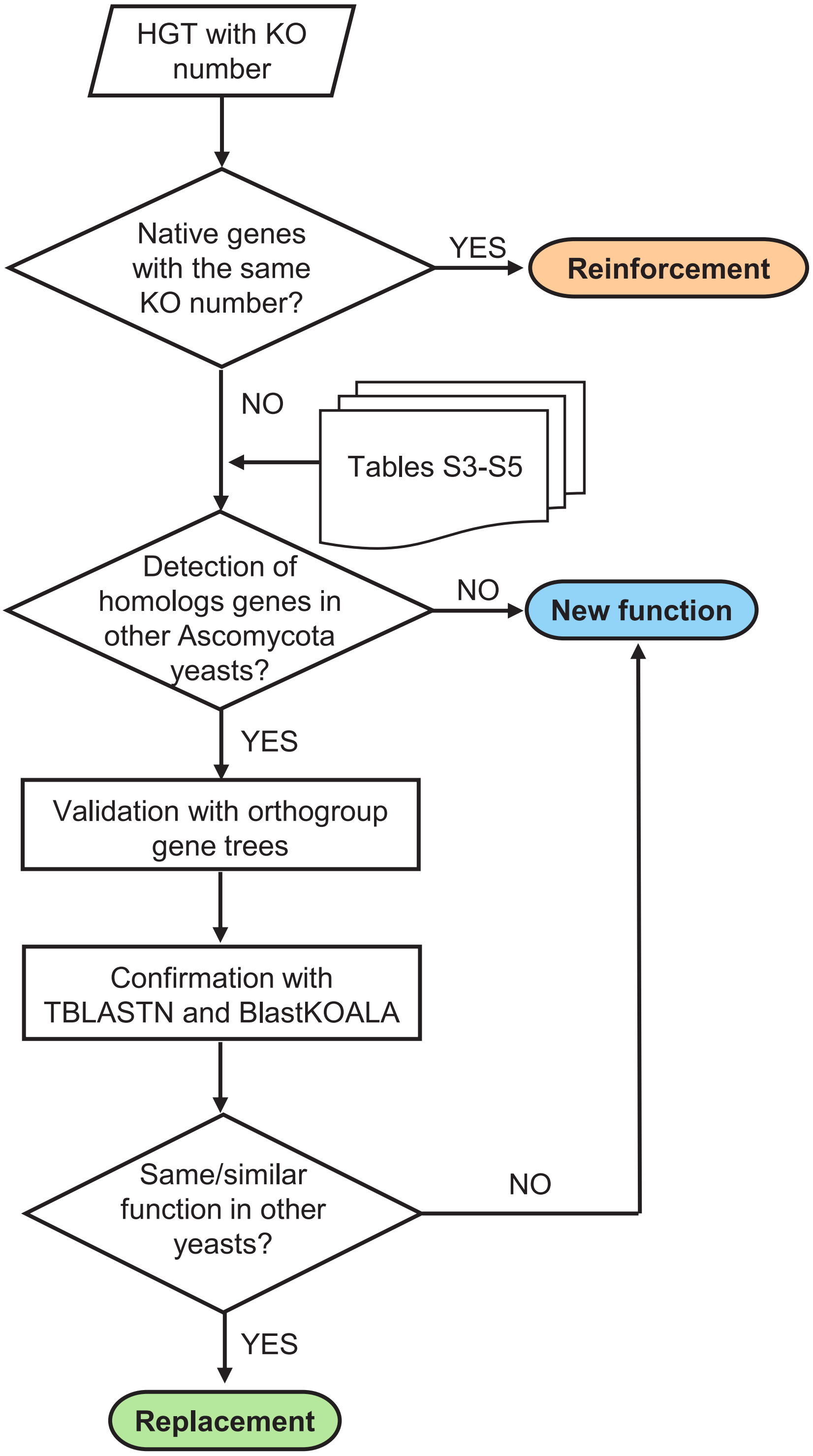

Fig. S5. Classification of horizontally acquired genes in their metabolic contribution to the host genome. Workflow diagram describing the strategy used to classify each acquired gene. Their contribution to the host genome was determined by evaluating the presence of homologous genes and their functions within Saccharomycotina subphylum.

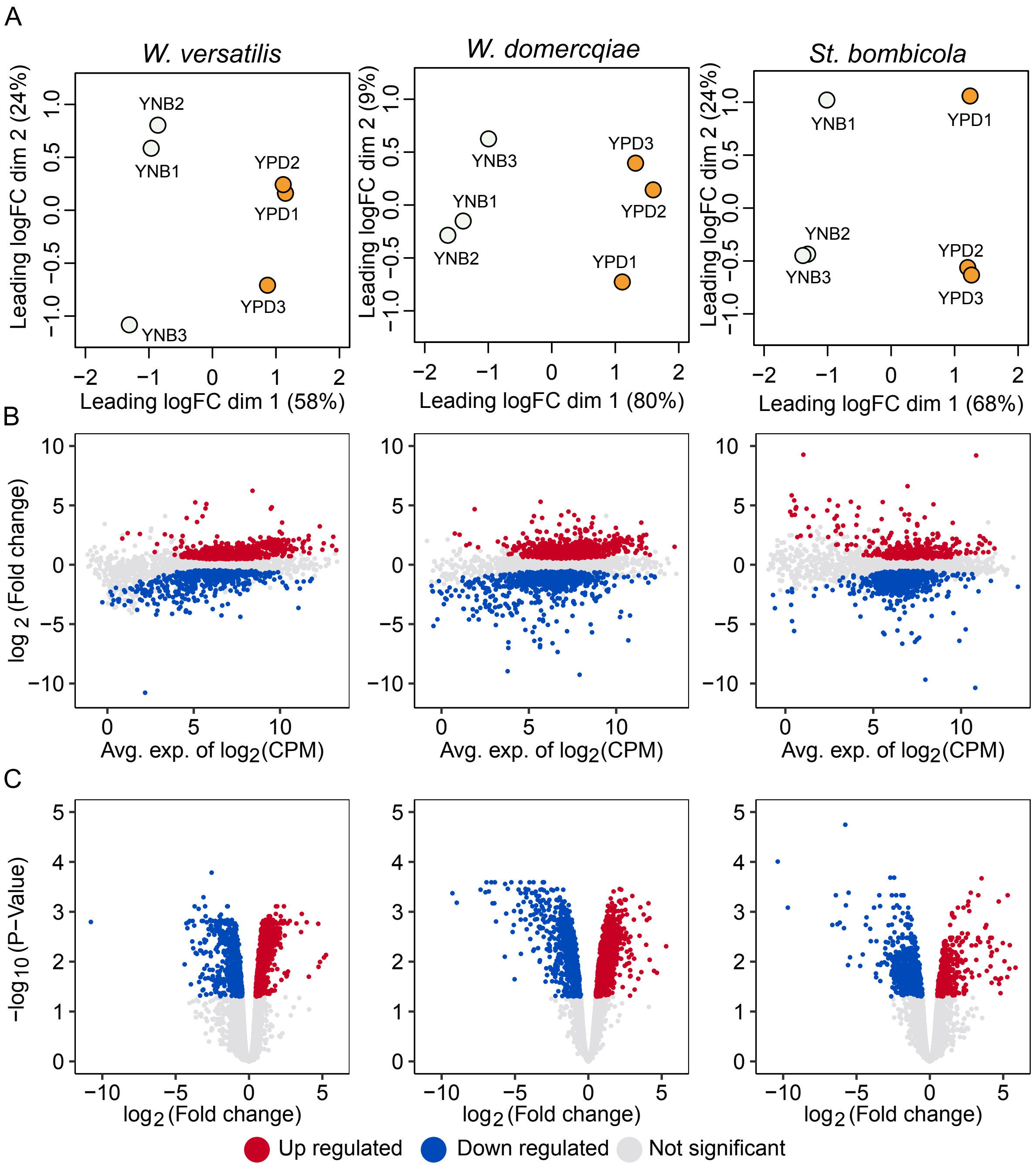

Fig. S6. Differential gene expression analysis between YPD and YNB media. Each column represents for each species, (A) multidimensional scaling (MDS), (B) mean-difference (MD) plot showing log-fold change and average expression level of each gene, and (C) volcano plots indicating the statistical significance in relation to the log-fold change expression. The two axes of MDS plots (top panels) represent the leading dimensions of variation in the log2FC space. The first principal dimension (X-axis) separates samples by medium, as expected, and accounts for the largest variance in the dataset (58%, 80%, 68%). The second dimension (Y-axis) captures remaining inter-replicate variability (24%, 9%, 24%), which is typical of RNA-seq experiments. No specific replicate behaves as an outlier; the observed variation reflects primarily natural biological noise.

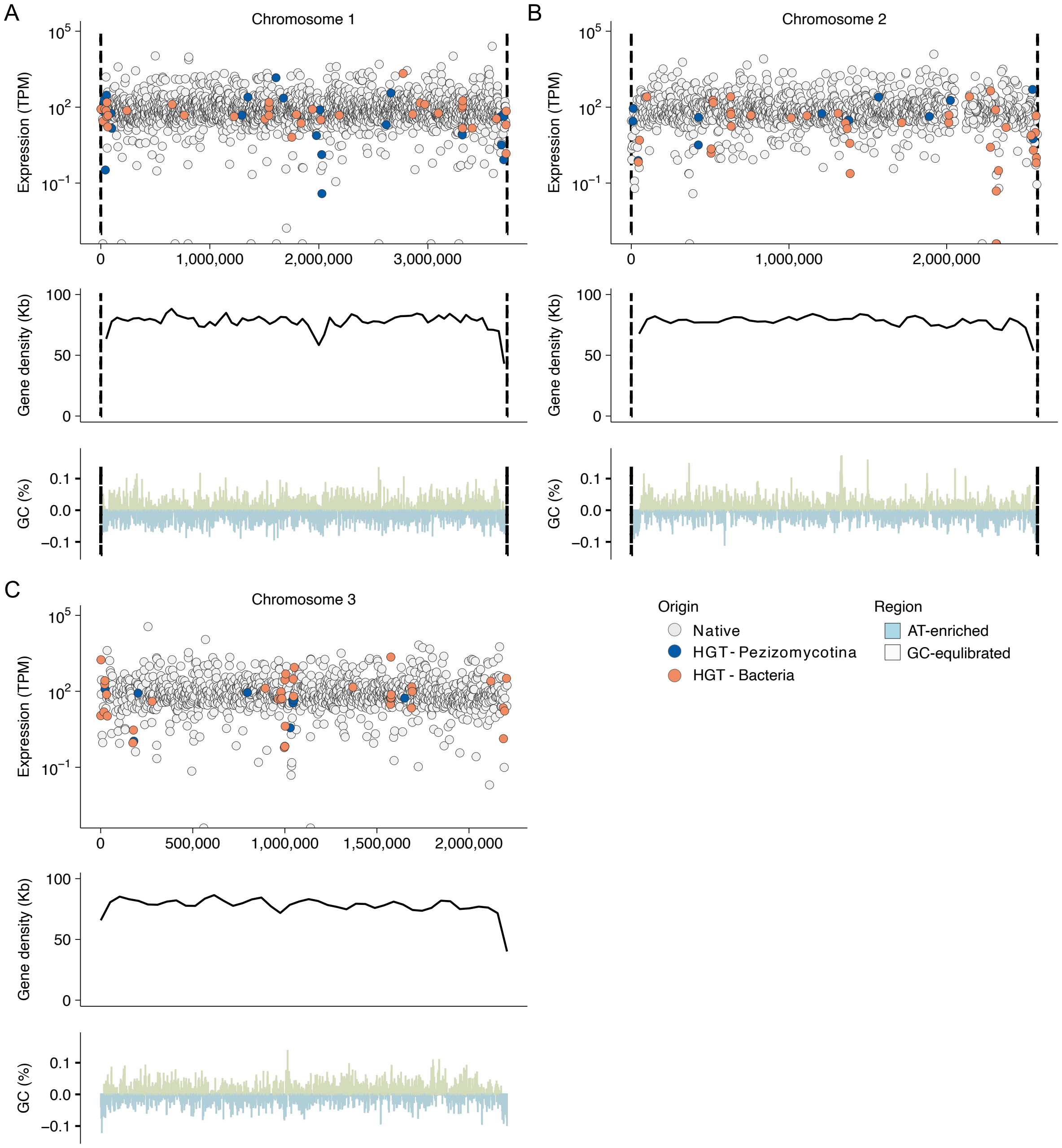

Fig. S7. Transcription levels *vs* chromosomal localization in *W. domercqiae*. Panels A, B and C depict identical analyses for nuclear chromosomes 1, 2 and 3 respectively. The top plot in each Panel shows for the pertinent chromosome the average transcription levels (y-axis) for native (grey) and horizontally acquired genes from Pezizomycotina (blue) and Bacteria (orange) plotted against chromosomal localization (x-axis). Vertical dashed lines mark telomere positions. Middle plots show gene density along chromosomes calculated as total genome length occupied by protein-coding genes in 100 kb sliding windows. Bottom panels display GC content as deviation from the genome average: GC-rich shown above (light green), and AT-rich below (light blue). Average expression levels in this figure are mean transcription levels in YPD and YNB media (three replicates each) measured in transcripts per million (TPM) and shown on a log_{10} scale. Full data is provided on Table S8 and Table "Source data".

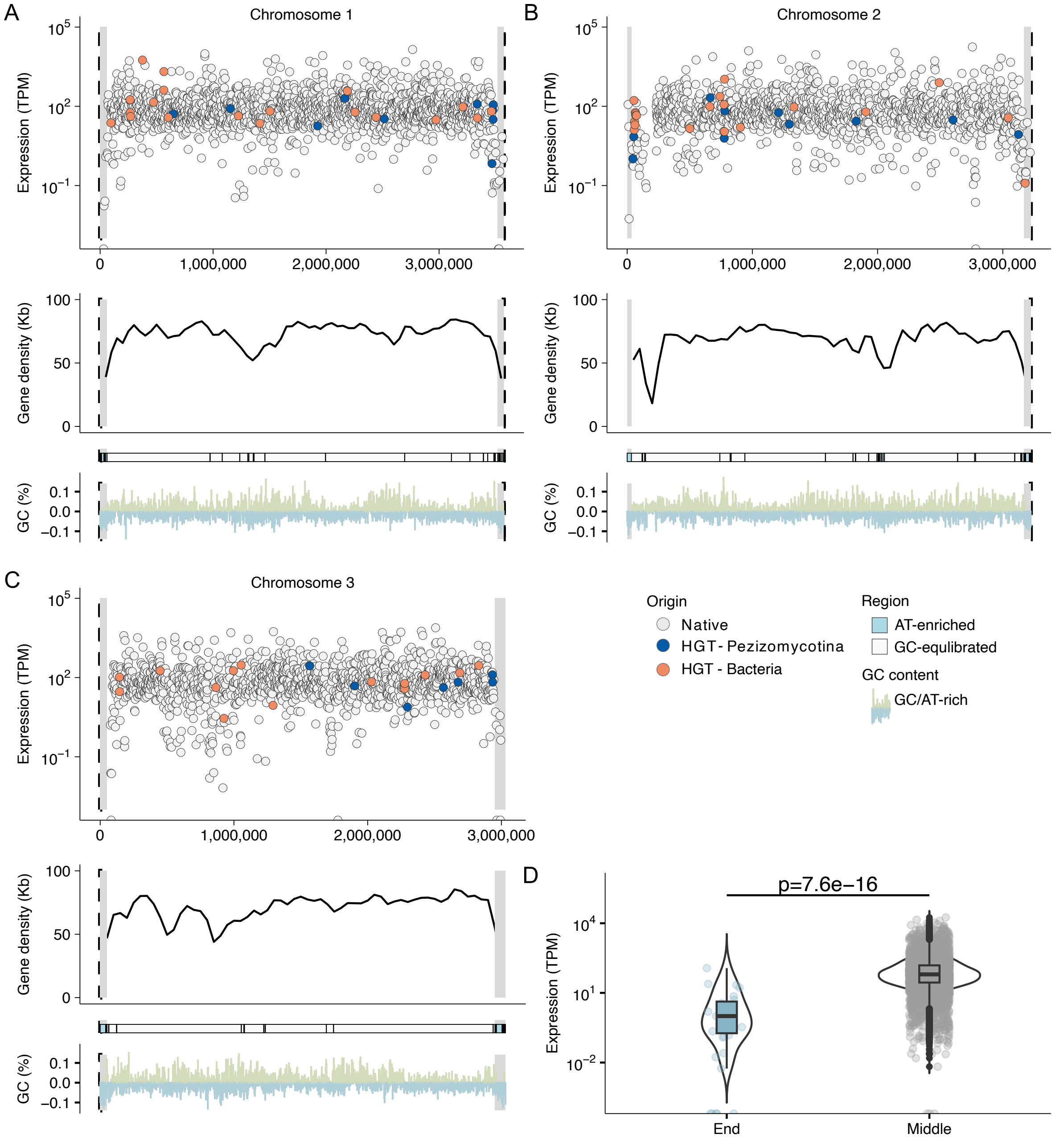

Fig. S8. Transcription levels *vs* chromosomal localization in *St. bombicola*. Panels A, B and C depict identical analyses for nuclear chromosomes 1, 2 and 3 respectively. The top plot in each Panel shows for the pertinent chromosome the average transcription levels (y-axis) for native (grey) and horizontally acquired genes from Pezizomycotina (blue) and Bacteria (orange) plotted against chromosomal localization (x-axis). Vertical dashed lines mark telomere positions. Middle plots show gene density along chromosomes calculated as total genome length occupied by protein-coding genes in 100 kb sliding windows. The horizontal bar shows the position of genomic regions classified as AT-rich (blue) or GC-equilibrated (white), by the software OcculterCut. Bottom panels display GC content as deviation from the genome average: GC-rich shown above (light green), and AT-rich below (light blue). Vertical grey bars at chromosome extremities mark the positions of "End" domains across all plots, determined based on the distribution of AT-rich regions identified with OcculterCut (full explanation of the determination of the localization and extent of "End" domains is given in Table S12). (D) violin plots depict average gene expression levels of all genes irrespective of

origin (native and xenologous of both origins), within “End” (blue) and “Middle” (grey) chromosomal domains. Statistical significance was assessed using the Mann-Whitney U Test ($U = 7675.5$, $p = 7.6x10-16$). For each gene the average expression level considered was the mean transcription levels calculated from six RNA-seq data sets (YPD and YNB grown cells, three replicates for each condition) measured in transcript per million (TPM) and plotted on a $\log_{10}$ scale. Data include genes from all three chromosomes. Full data is provided on Table S9 and Table “Source data”.

A

B

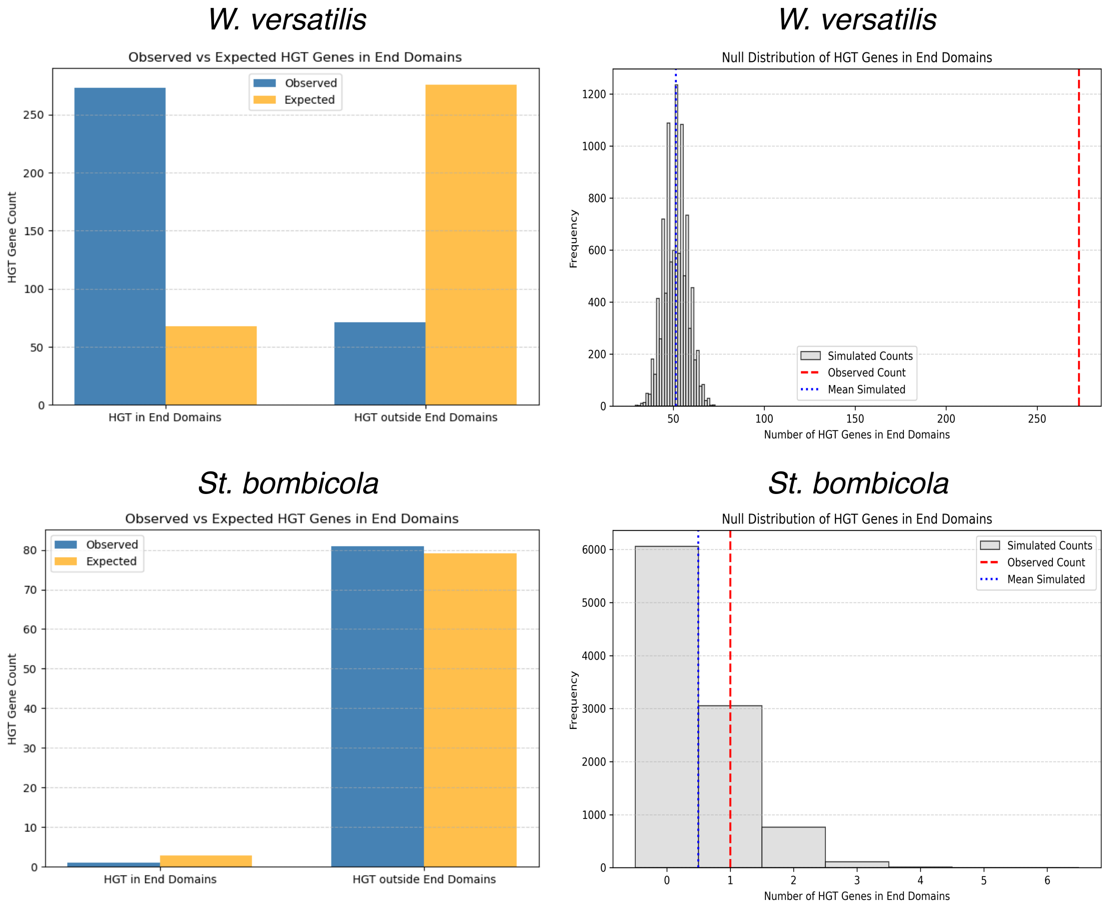

Fig. S9. Statistical assessment of xenologous (HGT) gene enrichment in "End" chromosomal domains in (A) *W. versatilis* and (B) *St. bombicola*. Two complementary approaches were applied to test for enrichment of xenologous genes within chromosomal "End" domains: (i) a chi-squared test where the expected number of HGT genes within the "End" domains, calculated based on the proportion of the genome occupied by those regions, was compared with the observed number, and (ii) a non-parametric permutation test where gene labels were randomly reassigned to known gene positions while preserving the total number of HGT and the same genomic structure and gene density across chromosomes. Both tests were congruent in their results indicating that **"End" chromosomal domains are significantly enriched by xenologous genes in *W. versatilis*** (chi-square=773.2, df=1, p-value=3.6x10^{-170}; empirical p-value after 10,000 iterations=0.00000, corresponding to 273 observed HGT in End domains and mean and standard deviation of simulated HGT counts in End domains of 51.4 and 6.4, respectively) **but not in *St. bombicola*** (chi-square=1.2, df=1, p-value=0.27; empirical p-value after 10,000 iterations=0.39, corresponding to 1 observed HGT in End domains and mean and standard deviation of simulated HGT counts in End domains of 0.5 and 0.7, respectively). Full data is provided on Table "Source data".

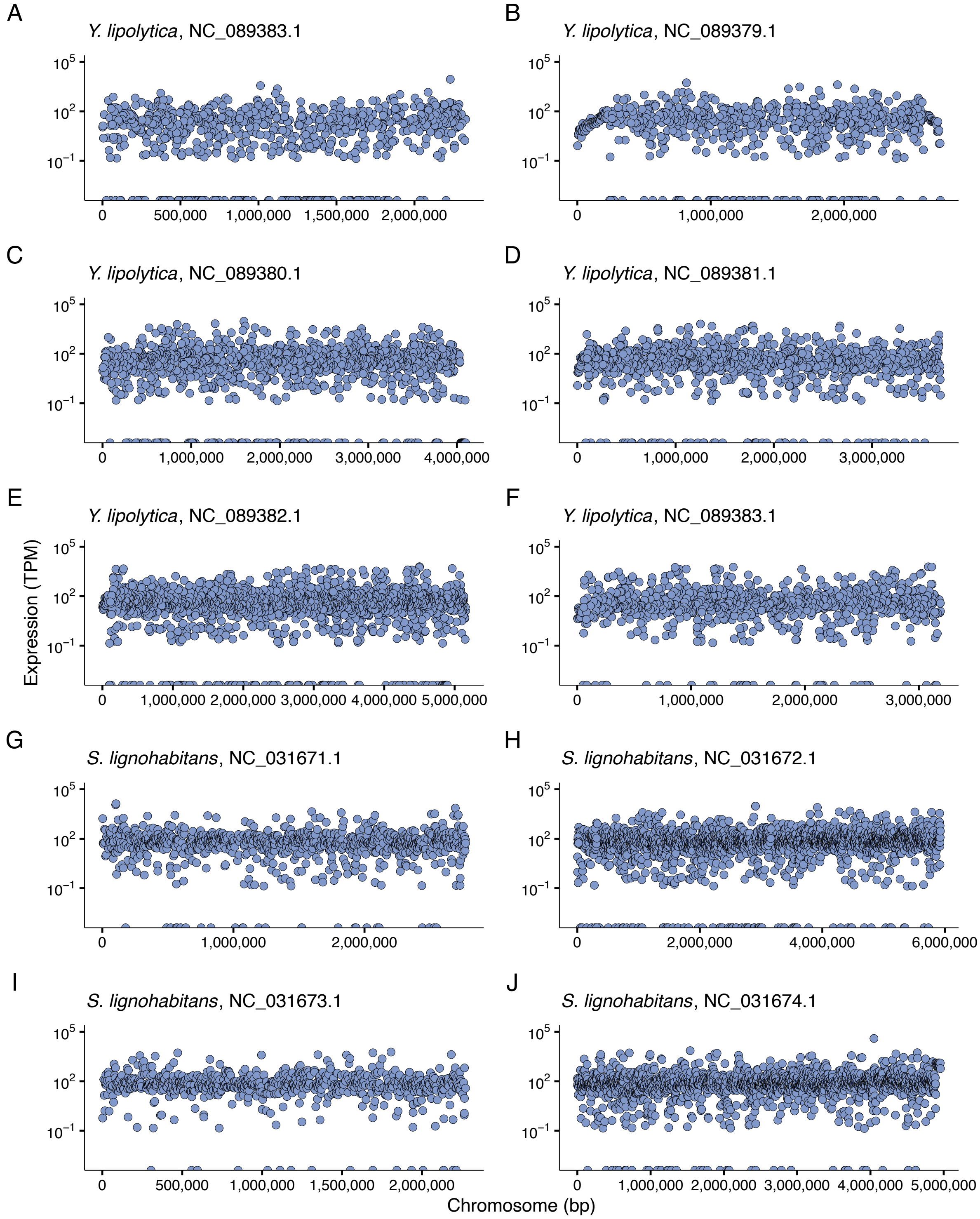
A
Y. lipolytica, NC_089383.1
B
Y. lipolytica, NC_089379.1
C
Y. lipolytica, NC_089380.1
D
Y. lipolytica, NC_089381.1
E
Y. lipolytica, NC_089382.1
F
Y. lipolytica, NC_089383.1
G
S. lignohabitans, NC_031671.1
H
S. lignohabitans, NC_031672.1
I
S. lignohabitans, NC_031673.1
J
S. lignohabitans, NC_031674.1
Expression (TPM)
Chromosome (bp)

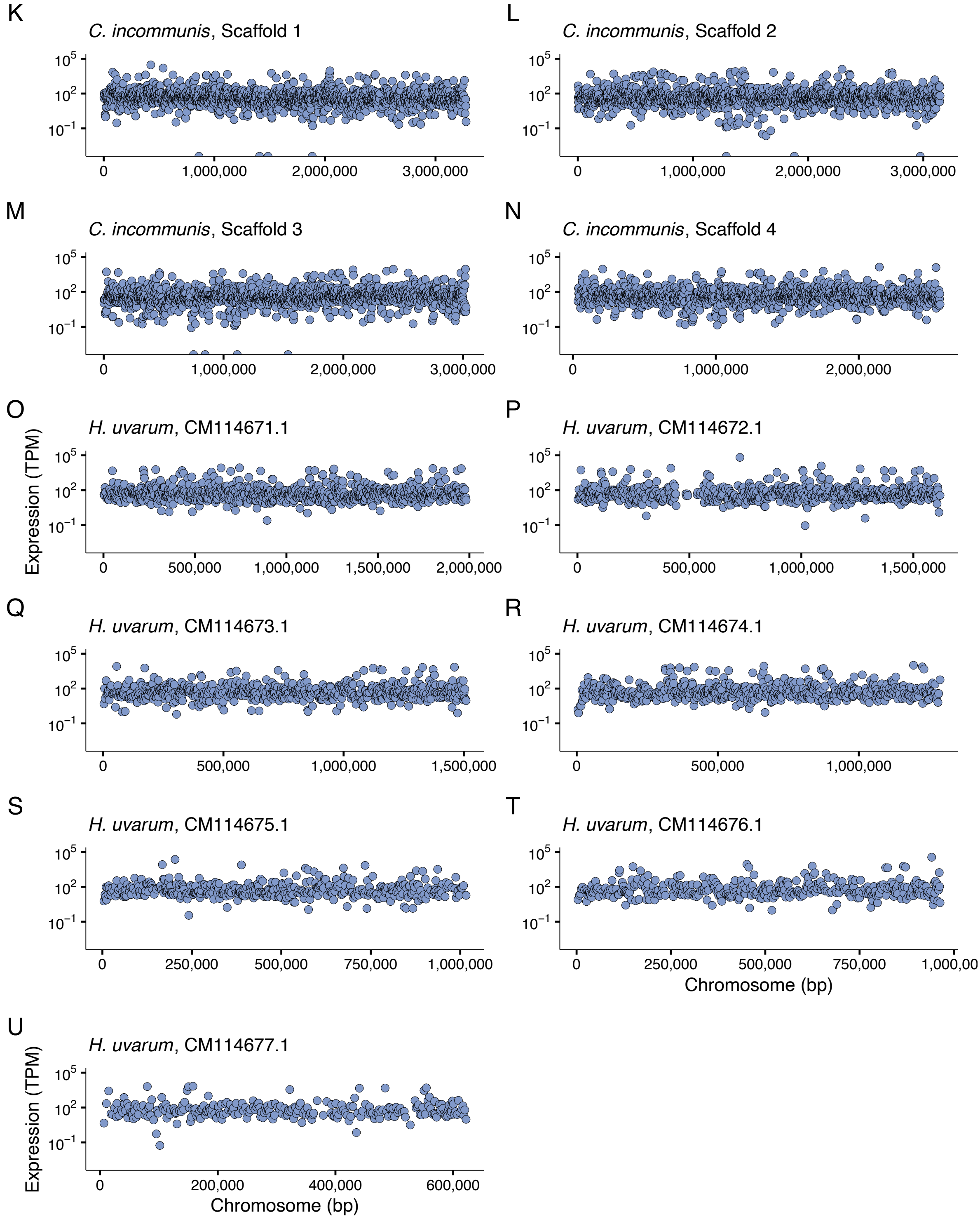

Fig. S10. Transcription levels *vs* chromosomal localization of outgroup species. (*Yarrowia lipolytica, Sugiyamaella lignohabitans*, *Candida incommunis*) and of *Hanseniaspora uvarum.* Distribution of transcription levels along the genome of *Y. lipolytica* DSM 3286 (A-F), *S. lignohabitans* CBS 10342 (G-J), *C. incommunis* PYCC 4837 (K-N), and *H. uvarum* NRRL Y-1614 (O-U). Data for *Y. lipolytica* and *S. lignohabitans* are average transcription levels of three replicates, while for *C. incommunis* and *H. uvarum* only one replicate was available. This plots are comparable to those presented in the top panels of Figs.6, S7 and S8. Full data is provided on Table "Source data".

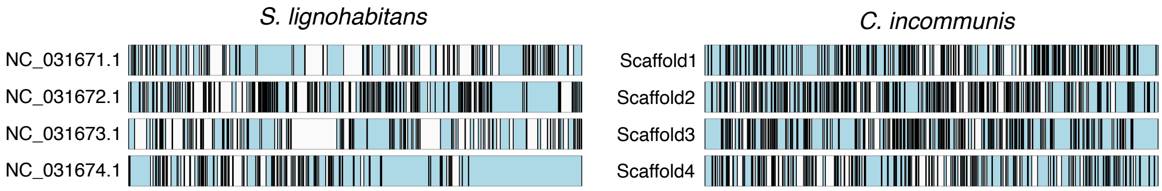

Fig. S11. Classification of genomic regions into AT-rich and GC-equilibrated segments using OcculterCut. The genomes of *Sugiyamaella lignohabitans* CBS 10342 and *Candida incommunis* PYCC 4837 were analyzed using OcculterCut, which identified GC-content thresholds of 45.0% and 46.7%, respectively. These thresholds were used to classify genome segments as either AT-rich or GC-equilibrated. Contiguous segments with the same classification were merged and plotted for each chromosome. Straight black lines indicate the boundaries between AT-rich (blue) from GC-equilibrated (white) regions. Chromosome/Scaffold names are shown on the left. The genomes of *Yarrowia lipolytica* DSM 3286, and *Hanseniaspora uvarum* NRRL Y-1614 were also analyzed with OcculterCut, but no GC-content threshold could be identified for these species, therefore, domains could not be defined in these cases. Full data is provided on Table "Source data".

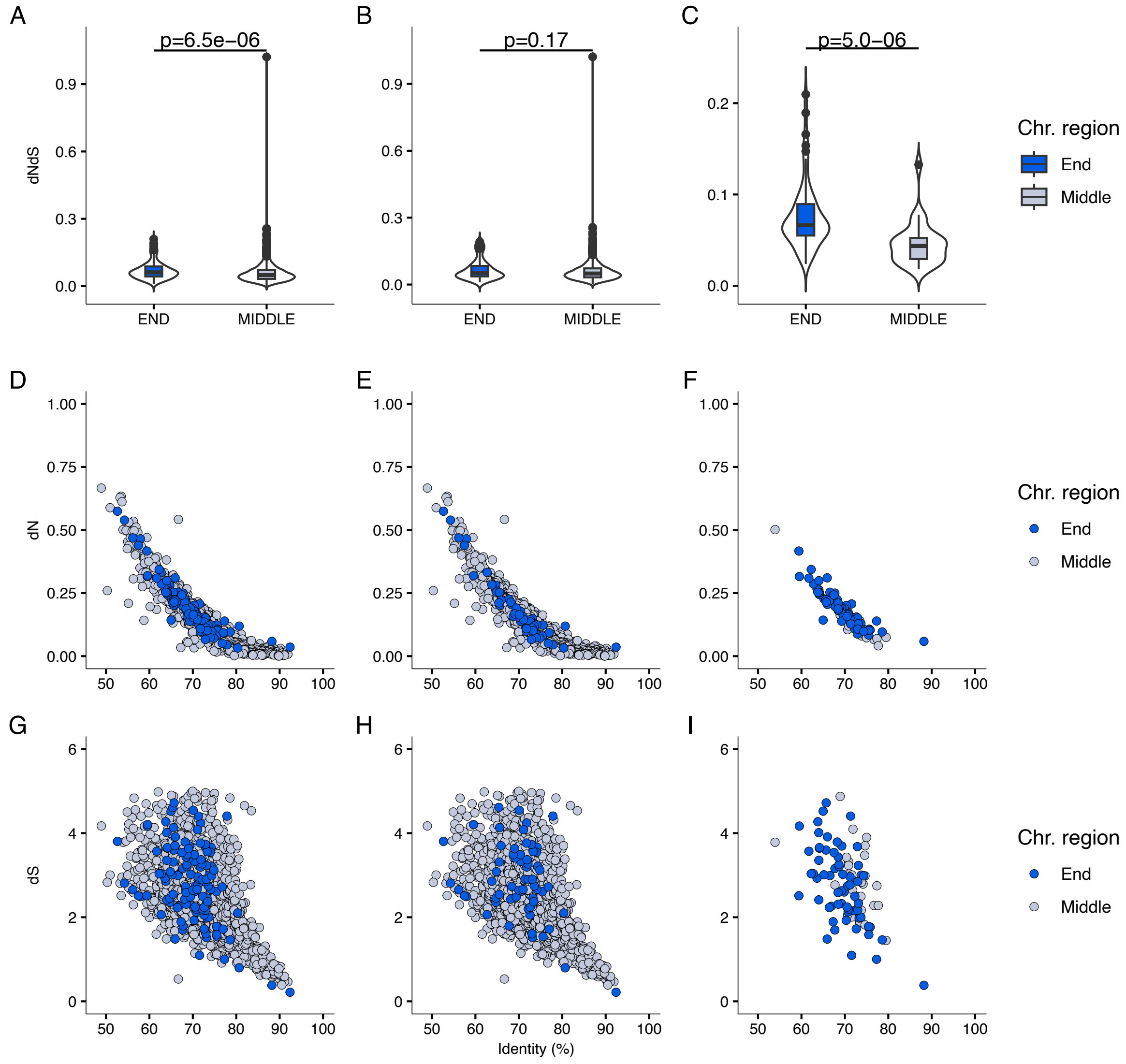

Fig. S12. Rates of synonymous and non-synonymous substitutions for native and xenologous genes between *W. versatilis* and a closely related *Wickerhamiella* species. Distribution of dN/dS ratio (A-C) for genes located in "End" and "Middle" chromosomal regions, showing that elevated evolutionary rate in "End" domains (A) are mainly due the contribution of xenologous genes (C), since for native genes (B) no statistically significant differences were found. (D-I) Rates of non-synonymous (dN) and synonymous (dS) substitutions plotted against percentage of gene identity measured at the nucleotide level. From left to right plots contain results from all genes (A, D, G), from native genes (B, E, H) and from xenologous genes (C, F, I). P-values are significative values that resulted from Mann-Whitney U tests performed to test for significant differences between dN/dS ratios of genes located in "End" and "Middle" regions considering all genes (U=205411, $p=6.5x10^{-6}$), only native (U = 90658, p=0.17) and xenologous genes (U = 1012, $p=5.0x10^{-6}$). Results include genes localized in all three chromosomes. Full data is provided on Table "Source data".

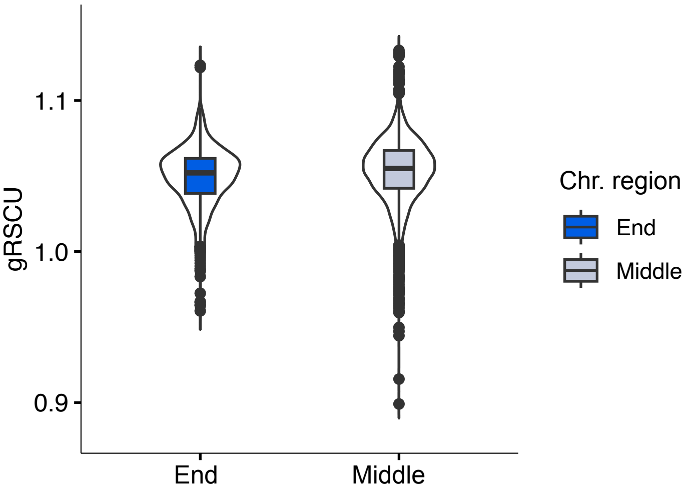

Fig. S13: Gene-wise relative synonymous codon usage in *W. versatilis* genes. Relative synonymous codon usage was estimated for each gene as the mean ratio of the observed frequency of synonymous codons to the expected frequency when all synonymous codons are equally used. Color code indicates gene localization in "End" and "Middle" chromosomal regions**.** Results include genes from all three chromosomes. Full data is provided on Table "Source data".
