# Supplementary material for: Regions of low gene expression promote maintenance and adaptation of horizontally acquired genes in yeasts
Source: Commun Biol. 2026 May 9;9:968. doi: 10.1038/s42003-026-10153-8 (PMC13376923; doi:10.1038/s42003-026-10153-8)
Supplement: Supplementary file 2 — Description of Additional Supplementary Files [file 42003_2026_10153_MOESM2_ESM.doc]

**SI Guide for:**

**Regions of low gene expression promote maintenance and adaptation of horizontally acquired genes in yeasts**

Patrícia H. Brito^1,2*^, Victoria Gil^1,2^, Ana Pontes^1,2^, Margarida Silva^1,2^, Carla Gonçalves^1,2^ and Paula Gonçalves^1,2*^

* Corresponding authors

Email: phbrito@fct.unl.pt Email: pmz@fct.unl.pt

^1^ Associate Laboratory i4HB - Institute for Health and Bioeconomy, NOVA School of Science and Technology, Universidade NOVA de Lisboa, 2829-516 Caparica, Portugal.

^2^ UCIBIO - Applied Molecular Biosciences Unit, Department of Life Sciences, NOVA School of Science and Technology, Universidade NOVA de Lisboa, 2829-516 Caparica, Portugal.

**This PDF file includes information on all supplementary files:**

- **Supplementary Figures**

This file contains supplementary Figures S1 to S13.

- **Supplementary Data 1**

Taxonomy, strain ID, and source information of 118 fungi proteomes used in the OrthoFinder analysis.

- **Supplementary Data 2**

Orthogroup composition. Matrix generated by OrthoFinder that indicates the proteome sequences included in each orthogroup.

- **Supplementary Data 3-5**

This file contains Supplementary Data 3, Supplementary Data 4 and Supplementary Data 5. Orthofinder results and classification of genes' origin (“HGT-bacteria”, “HGT-Pezizomycotina”, and “not HGT-Pezizomycotina”) for the three W/S-clade species.

- **Supplementary Data 6-8**

**RNA-seq gene expression analysis of** the three W/S-clade species (*Wickerhamiella versatilis***,** *Wickerhamiella domercqiae*, and *Starmerella bombicola*). Results of transcript quantification in transcript per million (TPM), differential expression analysis, functional annotation performed with PANTHER and BlastKOALA, and classification of xenologous genes in their metabolic contribution to the host genome.

- **Supplementary Data 9**

Functional classification of the whole proteome of W/S-clade species discriminated for native and xenologous genes. Analyses were performed with PANTHER Classification System, PANTHER GO-Slim and PANTHER Protein Class annotations. Only top scoring functional inferences with Evalue < 10^-6^ are included.

- **Supplementary Data 10.**

Functional annotation of xenolog genes from the W/S-clade species. Comparison between BlastKOALA, PANTHER, EggNOG, and InterProScan annotation pipelines.

- **Supplementary Data 11-12**

Mapping results of RNA-seq reads to the reference genomes, and summary information of (1) the reference genomes used in RNA-seq analysis, (2) Nanopore assemblies of reference genomes, and (3) localization of the telomeres.

- **Supplementary Data 13**

**Approximate boundaries of chromosomal "End" and "Middle" regions, defined based on the AT-rich/GC-equilibrated segments in *W. versatilis* and *St. bombicola* genomes.** The following four excel sheets present the raw data used to determine these chromosomal regions. "End" domains are represented by blue bars on Figures 6A-C and S8A-C.

- **Supplementary Data 14**

Transposon elements (TE). Prediction and annotation performed using Earl Grey TE annotation pipeline.

- **Supplementary Data 15**

Summary information and accession numbers of assemblies and transcriptomes analyzed for outgroup species.

- **Supplementary Data 16**

Genome base composition estimated for the genomes of three W/S clade outgroup species using OcculterCut. On the left are the raw data and on the right are the distribution plots of GC genome GC content.

- **Supplementary Data 17**

Results from selection analyses performed on genes located at chromosomal ends. The test M1a vs M0 was performed on all alignments. Significant results after Bonferroni correction were tested for positive selection using tests M2a vs M1a and M8 vs M7. Log likelihood inferences were performed with codeml from PAML package.

- **Supplementary Data 18**

Total number of orthogrops per species and number of orthogroups that were automatically classified as not containing a gene acquired from a Pezizomycotina taxa (Not-HGT Pezizo), as well as the number of orthogroups that were manually analyzed by inspection of gene trees and BLASTp searches against NCBI nr database (Manually).

- **Supplementary Data 19**

SourceData – numerical source data for all main and supplementary charts/graphs.

- **Supplementary Data 20**

ZIP archive containing TE consensus libraries and GFF annotation files obtained for each genome assembly.

- **Supplementary Data 21**

ZIP archive containing proteins sequences annotated as DUF3435 (captain) and DUF3723 used to build the HMMER profiles for Starship transposon searches. These sequences were retrieved from Gluck-Thaler et al. (2022).

**Other supporting materials for this manuscript include custom-made scripts, supplementary tables, Nanopore assemblies and BUSCO ortholog lists that were deposited at Fishare repository:**

- Supplementary tables: <https://doi.org/10.6084/m9.figshare.29924882>
- Custom-made scripts: <https://doi.org/10.6084/m9.figshare.26527765>
- NCBI submissions of Nanopore assemblies and BUSCO ortholog lists: <https://doi.org/10.6084/m9.figshare.30085981>
